# The Korea-United States Air Quality (KORUS-AQ) field study

**DOI:** 10.1525/elementa.2020.00163

**Published:** 2021-05-12

**Authors:** James H. Crawford, Joon-Young Ahn, Jassim Al-Saadi, Limseok Chang, Louisa K. Emmons, Jhoon Kim, Gangwoong Lee, Jeong-Hoo Park, Rokjin J. Park, Jung Hun Woo, Chang-Keun Song, Ji-Hyung Hong, You-Deog Hong, Barry L. Lefer, Meehye Lee, Taehyoung Lee, Saewung Kim, Kyung-Eun Min, Seong Soo Yum, Hye Jung Shin, Young-Woo Kim, Jin-Soo Choi, Jin-Soo Park, James J. Szykman, Russell W. Long, Carolyn E. Jordan, Isobel J. Simpson, Alan Fried, Jack E. Dibb, SeogYeon Cho, Yong Pyo Kim

**Affiliations:** 1 NASA Langley Research Center, Hampton, VA, USA; 2 Air Quality Research Division, National Institute of Environmental Research, Incheon, Republic of Korea; 3 National Center for Atmospheric Research, Boulder, CO, USA; 4 Yonsei University, Seoul, Republic of Korea; 5 Hankuk University of Foreign Studies, Seoul, Republic of Korea; 6 Seoul National University, Seoul, Republic of Korea; 7 Konkuk University, Seoul, Republic of Korea; 8 Ulsan National Institute of Science and Technology, Ulsan, Republic of Korea; 9 Inha University, Incheon, Republic of Korea; 10 Kum Kyoung Engineering, Seoul, Republic of Korea; 11 NASA Headquarters, Washington, DC, USA; 12 Korea University, Seoul, Republic of Korea; 13 University of California, Irvine, CA, USA; 14 Gwangju Institute of Science and Technology, Gwangju, Republic of Korea; 15 US Environmental Protection Agency, Office of Research and Development, Research Triangle Park, NC, USA; 16 National Institute of Aerospace, Hampton, VA, USA; 17 University of Colorado, Boulder, CO, USA; 18 University of New Hampshire, Durham, NH, USA; 19 Ewha Womans University, Seoul, Republic of Korea

**Keywords:** KORUS-AQ, Seoul, Air quality, Ozone, PM2.5, Transboundary pollution

## Abstract

The Korea–United States Air Quality (KORUS-AQ) field study was conducted during May–June 2016. The effort was jointly sponsored by the National Institute of Environmental Research of South Korea and the National Aeronautics and Space Administration of the United States. KORUS-AQ offered an unprecedented, multi-perspective view of air quality conditions in South Korea by employing observations from three aircraft, an extensive ground-based network, and three ships along with an array of air quality forecast models. Information gathered during the study is contributing to an improved understanding of the factors controlling air quality in South Korea. The study also provided a valuable test bed for future air quality–observing strategies involving geostationary satellite instruments being launched by both countries to examine air quality throughout the day over Asia and North America. This article presents details on the KORUS-AQ observational assets, study execution, data products, and air quality conditions observed during the study. High-level findings from companion papers in this special issue are also summarized and discussed in relation to the factors controlling fine particle and ozone pollution, current emissions and source apportionment, and expectations for the role of satellite observations in the future. Resulting policy recommendations and advice regarding plans going forward are summarized. These results provide an important update to early feedback previously provided in a Rapid Science Synthesis Report produced for South Korean policy makers in 2017 and form the basis for the Final Science Synthesis Report delivered in 2020.

## 1. Introduction

Air quality is an environmental concern of fundamental importance across the globe. The need to monitor and understand air quality requires continual effort as the landscape of emissions evolves in response to changes in population, energy use, and industrial activity. Air quality goals have also evolved with improved understanding of health effects, demonstrating the added benefit of setting lower targets for exposure of humans and ecosystems to ozone, fine particles, and other toxic pollutants in the air (e.g., World Health Organization [WHO] 1987, 1996, 2000, 2006; Bachmann 2007). Historically, efforts to diagnose regions of poor air quality have relied primarily on ground-based observations along with modeling to develop mitigation strategies. In recent years, satellites in low Earth orbit (LEO) have demonstrated the ability to observe the critical constituents affecting air quality. However, the impact of LEO satellites has been limited due to their frequency of observation (approximately once per day) and coarse resolution (horizontal scales of tens of km) that cannot resolve the source distributions and chemical evolution controlling air quality conditions that develop over timescales of a single day. This has recently improved with the launch of the TROPOspheric Monitoring Instrument, providing unprecedented resolution (3.5 × 75.5 km^2^) with daily global coverage. This enables the identification of specific point sources and fine-scale urban source distributions (Beirle et al., 2019; Goldberg et al., 2019a). Geostationary (GEO: geosynchronous equatorial orbit) observations are needed to provide the additional requirement for more continuous observations over the course of the day as emissions, transport, and chemistry evolve. The drawback of GEO is the limited viewing domain, preventing global observations with a single satellite. This has led to an international effort to launch a constellation of satellite instruments focused on air quality over the major population centers of the northern hemisphere: Asia, North America, and Europe. These GEO instruments will provide hourly observations of those regions throughout the day at horizontal resolutions of better than 10 km.

The constellation approach requires a commitment to strong collaboration, especially regarding common strategies for satellite calibration and validation. Joint work on the interpretation of satellite observations and best practices for integrating information from satellites with more traditional ground monitoring is also needed. This need for collaboration played a key role in the genesis of the Korea–United States Air Quality (KORUS-AQ) field study. The study was jointly sponsored by the two organizations responsible for geostationary satellite observations of air quality over Asia and North America: the National Institute of Environmental Research (NIER) in South Korea and the National Aeronautics and Space Administration (NASA) in the United States. The collaboration also involved the broader atmospheric chemistry community by organizing a team of scientists from dozens of institutions across governmental, academic, and private sectors.

The decision to conduct the study in South Korea was based on several factors. Foremost was the recognition that South Korea faces air quality conditions that warrant urgent attention. Its location on the Asian Pacific Rim places it in a unique location where large gradients exist in atmospheric composition. The Korean peninsula is distinct, isolated by surrounding waters that offer the ability to assess upwind influences and downwind outflows under the right meteorological conditions. Interesting gradients also exist within South Korea. The city of Seoul and the surrounding metropolitan area occupies the northwest corner of the country. While only accounting for 12% of the land area, it is home to 25 million people or roughly half of the country’s population. Combined with the industrial activities and power plants along the northwest coast, a dominant fraction of emissions is contained in a relatively small area. This provided an ideal setting to examine air quality across megacity, country, and regional scales.

The timing of the study was focused on the pre-monsoon period from late spring into early summer (May–June). While pollution associated with fine particulate matter peaks earlier in the year during late winter and early spring, the pre-monsoon is associated with the most photochemically active period during which both ozone and fine particle pollution episodes can occur. The pre-monsoon also focuses more attention on the impact of local emissions since transboundary transport is maximized in the March–April timeframe.

The second section of this article provides an overview of the organization and execution of the KORUS-AQ study. The third section summarizes the air quality conditions observed in South Korea during the study and the associated meteorological conditions that are described in greater detail by Peterson et al. (2019). The fourth section of this article highlights major findings resulting from analyses of the KORUS-AQ observations and points to associated papers in this special issue of *Elementa* and other journals. These papers expand on the details regarding the factors controlling fine particulate matter and ozone pollution in South Korea, source apportionment as it relates to key pollution precursors, emissions verification, and air quality model assessment. The fifth section discusses KORUS-AQ observations in the context of expectations for future geostationary satellite observations and their incorporation into air quality assessments. The sixth section summarizes policy recommendations resulting from major findings, and the final section discusses the path forward in terms of prospects for future observational studies and analysis.

## 2. Study organization and execution

**Figure 1.**
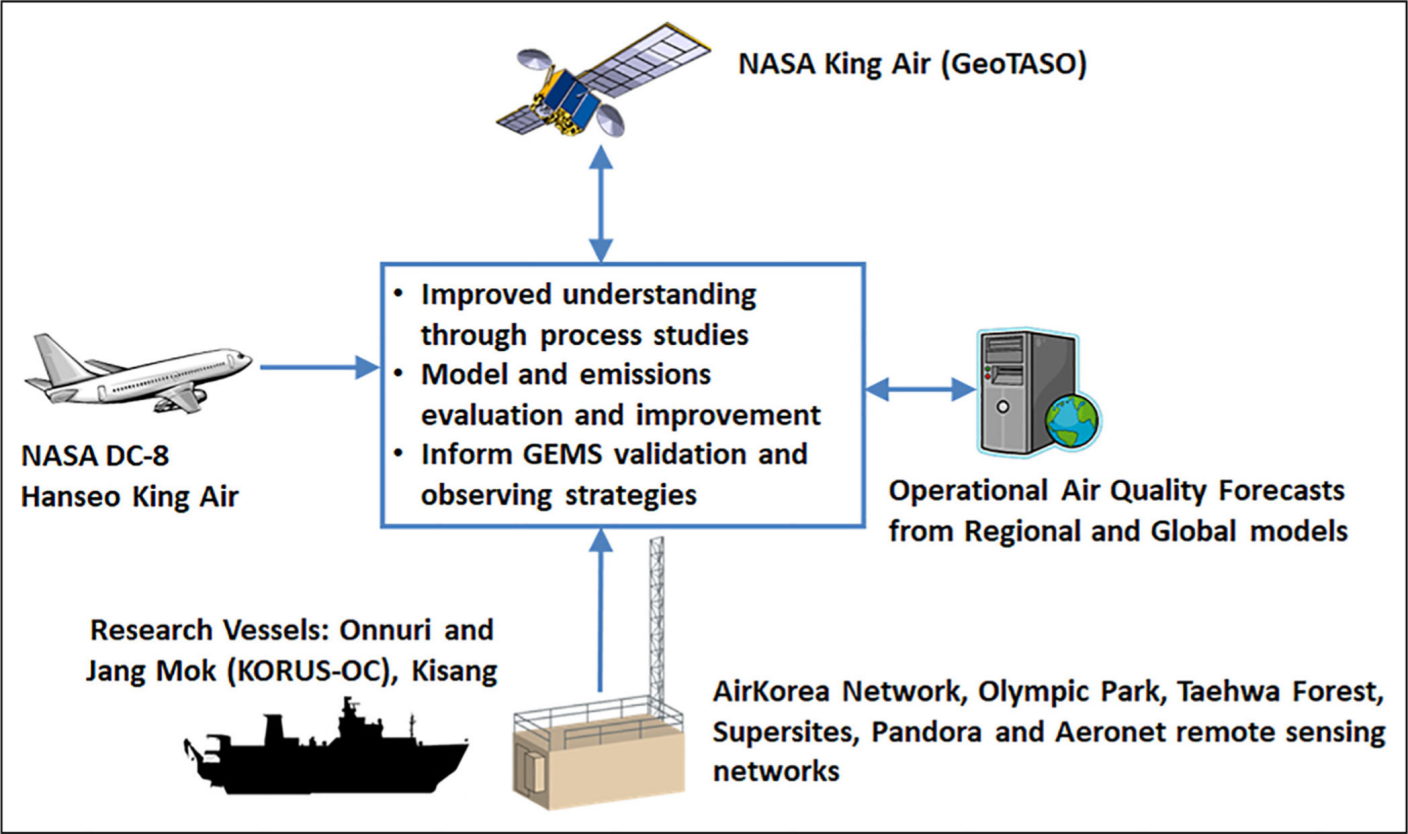
Schematic representation of the observing strategy used to address Korea–United States Air Quality science goals and explore the synergy between multi-perspective observations from the ground, air, and space. Details for each listed asset are provided in the text. DOI: https://doi.org/10.1525/elementa.2020.00163.f1

The KORUS-AQ study was conducted from May 2 to June 10, 2016. The study implemented a multi-perspective observing strategy intended to explore the synergy between operational air quality networks, air quality models, and geostationary satellite observations that will be realized with the recent launch of the Geostationary Environment Monitoring Spectrometer (GEMS) over Asia in February 2020 (Kim et al., 2020). This observing strategy is represented schematically in Figure 1 to show how the KORUS-AQ observational assets were deployed and used to address the fundamental science of air quality in South Korea, enable model evaluation and improvement, and contribute to the development of strategies for the validation and interpretation of GEMS observations. Intensive observations were collected from three aircraft, two comprehensive ground-based research sites, additional ground-based site augmentations, and three ships. A suite of air quality models was also employed to guide the collection of observations, particularly for the aircraft flight planning. Additional details on each of these components are provided below.

### 2.1. Observational assets

#### 2.1.1. Ground observations

**Figure 2.**
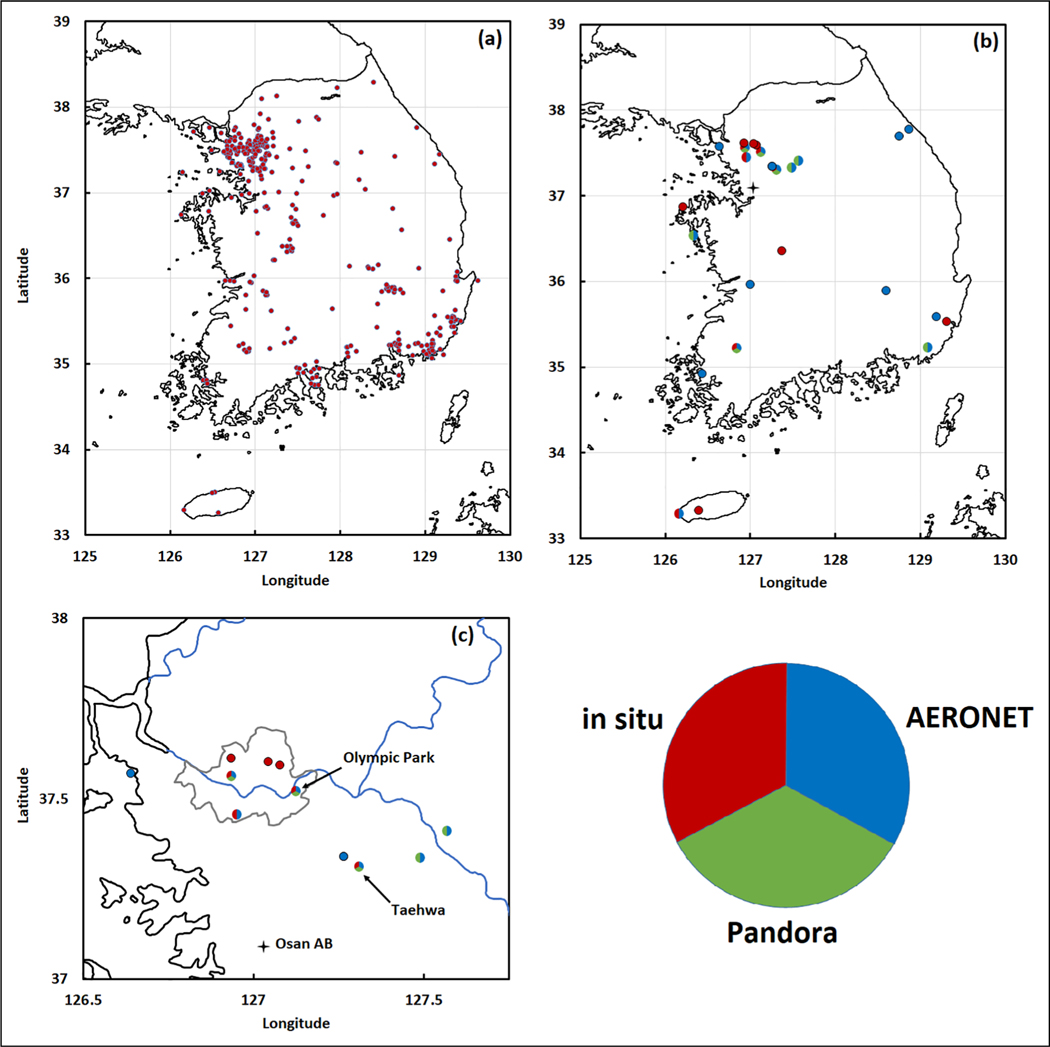
Ground-based observations during the Korea–United States Air Quality field study included (a) monitors comprising the AirKorea monitoring network and (b) research sites incorporating combinations of in situ observations (red), Pandora spectrometers (green), and AERONET sunphotometers (blue). Panel (c) offers an expanded view of sites located in the Seoul Metropolitan Area. Details on ground observations at these sites are provided in Tables 1–4. DOI: https://doi.org/10.1525/elementa.2020.00163.f2

The ground observations in support of KORUS-AQ provided a critical continuous record and direct determination of surface air quality conditions. The existing AirKorea monitoring network (https://www.airkorea.or.kr/eng) provided the anchor for KORUS-AQ observations. Organized under Korea’s Ministry of Environment and implemented by NIER, hourly observations of criteria pollutants including fine particulate matter and ozone are collected across the Korean peninsula from more than 300 instrumented sites (see Figure 2a). This operational monitoring system was supplemented by supersites at Olympic Park in Seoul and Taehwa Forest Research Station. Additional augmentation of ground measurements was implemented at numerous sites across the peninsula as well as Baengnyeong and Jeju islands (see Figure 2b). Networks of Pandora spectrometers and aerosol robotic network (AERONET) sunphotometers provided an additional element of ground-based remote sensing useful in understanding how to bridge the gap between surface in situ observations and remotely sensed quantities observed by satellites. Each of these ground-based augmentations is described in more detail below.

##### Olympic Park:

The Olympic Park site located south of the Han River and in the eastern part of the city provided an ideal location from which to monitor the details of air quality in Seoul (see Figure 2c). Operations at the site began on 8 May and continued through 17 June for the majority of instrument teams. This sampling window was offset from the flights, which started a week earlier, but the Olympic Park data collection period still encompassed 19 of the 23 days on which research flights were conducted. As will be discussed, this window also encompassed the most notable pollution events and shifts in air quality conditions encountered during the study. As the site with the most comprehensive characterization of atmospheric composition, it provided a focal point for much of the KORUS-AQ analysis and was the primary point of connection between surface and airborne observations. Details on the investigators, instruments, and observed quantities at Olympic Park are provided in Table 1.

##### Taehwa Research Forest:

Located approximately 30 km southeast of Olympic Park, Taehwa Research Forest provides a valuable contrast to conditions in Seoul (see Figure 2c). Nevertheless, it is still situated in the Seoul Metropolitan Area and is frequently impacted by pollution from Seoul and surrounding sources to the west of the site. Thus, this second most instrumented site sheds light on near-field downwind air quality impacts. Instruments at this site came online gradually during the first week of May and continued through the end of flight operations. The suite of measurements at Taehwa was focused more toward gas-phase composition, with particular emphasis on biogenic volatile organic compounds (VOCs) emitted at this heavily vegetated site. This location also benefitted from continuous information on air quality conditions related to ozone at the surface and aloft from the NASA TROPOZ differential absorption lidar. Similar to Olympic Park, this site provided an important link between surface and airborne measurements with frequent overflight by the research aircraft. Details on the investigators, instruments, and observed quantities at Taehwa Research Forest are provided in Table 2.

##### Additional ground site and ship-based observations:

In addition to the two main supersites, numerous ground sites across the Korean peninsula and island locations were augmented in support of the KORUS-AQ study (indicated by sites marked red in Figure 2b). This included six aerosol supersites maintained by NIER and university research sites. Particularly noteworthy observations include the active remote sensing at Seoul National University by the University of Wisconsin High Spectral Resolution Lidar (HSRL) and in situ submicron aerosol composition at the Korea Institute of Science and Technology (Kim et al., 2018). These measurements provided valuable comparisons with Olympic Park to assess when observations were representative of the greater Seoul area. Researchers also conducted measurements from three Korean research vessels: RVs Onnuri, Jang Mok, and Kisang. Coastal observations from the Onnuri and Jang Mok were conducted under a companion study on ocean color named KORUS-OC (Tzortziou et al., 2018; Thompson et al., 2019; Jordan et al., 2021a, 2021b). Details on the investigators, instruments, and observed quantities at these additional locations are provided in Table 3.

##### Ground-based remote sensing networks:

Ground-based remote sensing plays a critical role in validating satellite observations as well as relating column-integrated quantities to conditions at the surface. During KORUS-AQ, two networks were put in place. Remote sensing of aerosols was provided by AERONET sunphotometers (Holben et al., 2018), and trace gas observations were provided by Pandora spectrometers (Herman et al., 2018). These two networks are indicated by sites marked in blue (AERONET) and green (Pandora) in Figure 2b. Table 4 provides the coordinates for these sites. Ten AERONET sites were established in support of KORUS-AQ. Additional context was provided by nine more long-term AERONET sites in Korea as well as existing regional sites in surrounding regions/countries. The eight Pandora spectrometers were colocated alongside either temporary or long-term AERONET sunphotometers.

#### 2.1.2. Airborne observations

KORUS-AQ included three research aircraft that conducted flights from Osan Air Base located approximately 50 km south of Olympic Park (Figure 2c). The specific role of each platform is described below. Details on the investigators, instruments, and observed quantities for each aircraft are provided in Table 5.

##### NASA DC-8:

The largest of the three airborne platforms carried a comprehensive payload for in situ sampling of trace gas and aerosol composition. Remote sensing capability included measurements of actinic flux to diagnose photochemical rates, active remote sensing of ozone and aerosols above and below the aircraft, and passive remote sensing above the aircraft for aerosol optical depth and trace gas abundances of water vapor, ozone, and NO_2_.

##### Hanseo King Air:

This smaller in situ platform carried a payload to measure the key subset of trace gases that can be remotely sensed by satellites and ground-based remote sensors: ozone, NO_2_, SO_2_, CO, CH_2_O, CH_4_, CO_2_, and water vapor.

##### NASA King Air:

This aircraft was dedicated specifically to remote sensing of NO_2_, CH_2_O, SO_2_, and ozone. Flying above the ground sites and in situ aircraft in the study region, it provided an analog for the satellite observations of air quality that can be anticipated from geostationary satellites.

In addition to these three aircraft, another aircraft flew over the North China Plain in the vicinity of Xingtai in Hebei province during the same period. The study, called Air Chemistry Research in Asia, collected observations from a turboprop Y-12 airplane for an important upwind source region (Wang et al., 2018). Observations included aerosol optical properties (scattering and absorption), black carbon, trace gases (ozone, SO_2_, NO, NO_2_, NO_y_, CO, CH_4_, CO_2_, and H_2_O), and VOC grab samples.

#### 2.1.3. Satellite observations

The KORUS-AQ team leveraged information from existing satellites that played a critical role in providing wide area observations to help interpret surface and airborne observations in conjunction with synoptic weather patterns. The specific role of each platform is described below.

##### Geostationary Ocean Color Imager (GOCI):

GOCI provided information on aerosol optical properties including aerosol optical depth (AOD), fine mode fraction, single scattering albedo, and aerosol type.

##### Advanced Himawari Imager (AHI):

AHI is a geostationary meteorological satellite providing key context regarding weather patterns affecting KORUS-AQ observations as well as aerosol optical properties similar to GOCI.

##### Ozone Monitoring Instrument (OMI):

OMI provided daily observations of atmospheric pollutants (O_3_, NO_2_, SO_2_, CH_2_O, and aerosol properties) in the early afternoon from LEO using a UV-visible hyperspectral instrument. OMI data served as a proxy for the information that GEMS will provide with higher temporal and spatial resolution.

### 2.2. Execution of research flights

**Figure 3.**
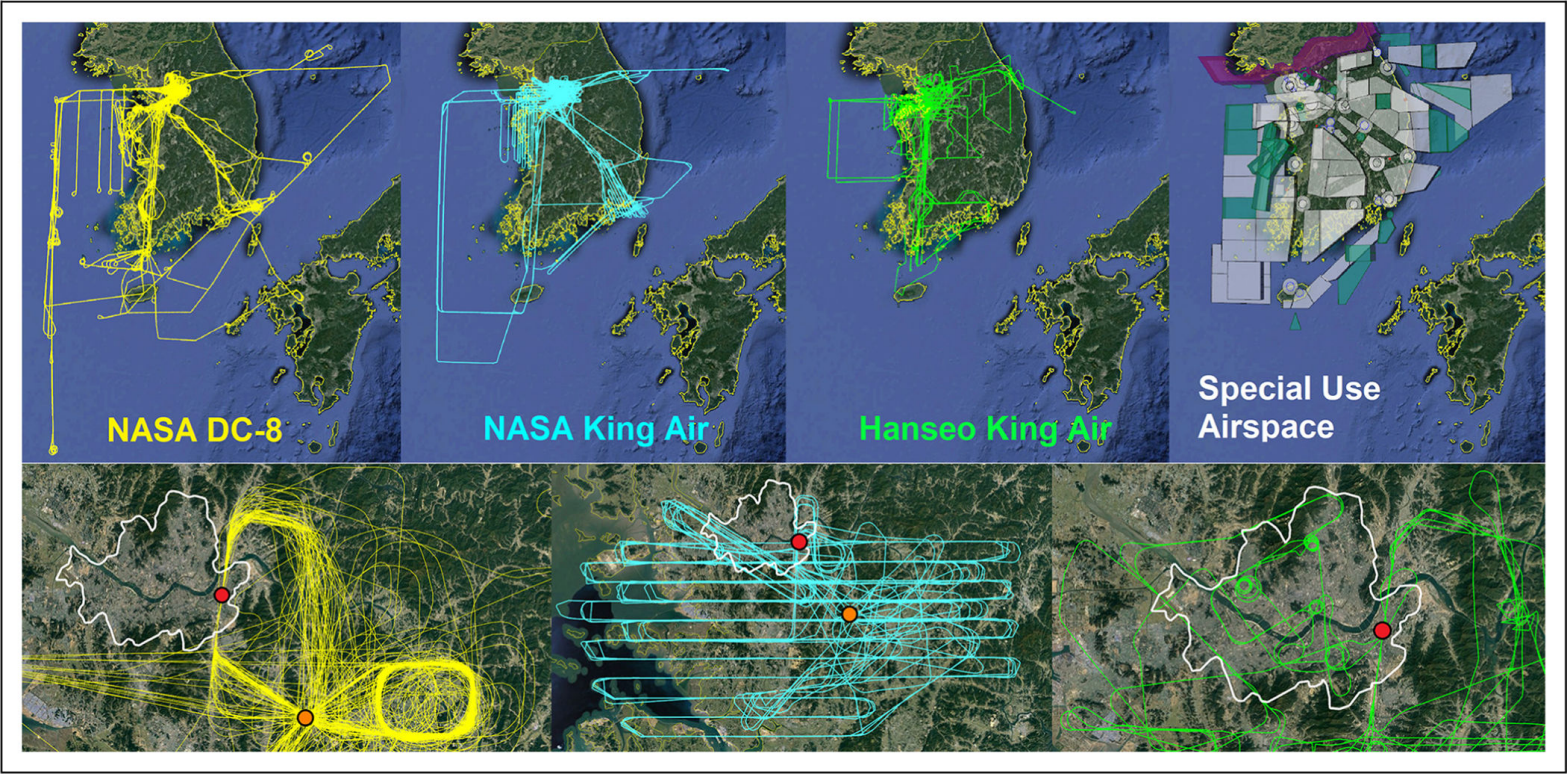
Google Earth images are overlaid with flight tracks for each of the Korea–United States Air Quality aircraft. Special Use Airspace affecting flight access is overlaid with circles representing airports and polygons representing military operations areas (white), restricted areas (teal), and prohibited areas (magenta). Expanded views of flight patterns conducted over the Seoul Metropolitan Area by each aircraft are shown in the bottom images with the Seoul City Boundary (white line) and research sites at Olympic Park (red) and Taehwa Forest (orange) marked. DOI: https://doi.org/10.1525/elementa.2020.00163.f3

During KORUS-AQ, flights were conducted on 23 days during the 40-day period from May 2 to June 10. With only a few exceptions, most flight days included all three aircraft. Flight tracks for each aircraft are shown in Figure 3. These flight patterns required detailed negotiation in advance and constant coordination during their execution. Daily flight planning was led by an array of teams producing air quality model forecasts and meteorological forecasters providing the necessary context to determine the best days and flight paths for sampling. Details on the investigators, specific models, and resolutions are provided in Table 6.

The need for careful planning and the location of flight lines can be better understood in the context of the Special Use Airspace over South Korea also shown in Figure 3. The polygons covering the majority of the peninsula and coastal waters are reserved for military training. Navigation of this complicated airspace was only possible by flying along the jetways connecting the airports (shown by circular airspace). Major jetways used most often during KORUS-AQ extended from Seoul to the south toward Jeju island, the southeast toward Busan and Pohang, the east toward Gangneung, and west to the Yellow Sea where north-south sampling could be conducted beyond the Special Use Airspace.

Special consideration was needed for sampling over Seoul and the surrounding metropolitan area. Here, airspace was even more restrictive with a prohibited area extending along the border with North Korea and covering Seoul north of the Han River. To enable routine overflight of the supersites at Olympic Park and Taehwa Research Forest, a stereoroute (repeated flight pattern) was established for the DC-8. As shown in Figure 3, the stereoroute was conducted under the full control of South Korea’s military air traffic authorities. The route would begin by guiding the DC-8 north of the Han River before turning to fly south starting at an altitude of approximately 3,000 ft. The DC-8 would then be directed to descend, reaching approximately 2,000 ft over the Han River, approximately 1,000 ft over the Olympic Park supersite, and finishing at less than 100 ft over the runway of Seoul Air Base. The DC-8 would then turn to the southeast and proceed at approximately 1,000 ft to overfly the Taehwa Research Forest supersite before initiating a spiral ascent to the east of Taehwa reaching up to approximately 7 km. Whenever possible the stereoroute was conducted at the start, middle, and finish of each DC-8 flight. In all, 55 descents over Olympic Park and 53 spirals east of Taehwa Research Forest were completed during the course of the study.

Between stereoroutes, the DC-8 most often conducted sampling along one of the jetways. This would begin with high altitude sampling at approximately 7 km altitude using the Differential Absorption Lidar and High Spectral Resolution Lidar (DIAL/HSRL) instrument to map the distribution of ozone and aerosols below the aircraft along the jetway. This information would be used to guide in situ sampling at lower altitudes along the jetway. Sampling along the length of the jetway would be conducted three more times with an initial return along the jetway at 1,000 ft followed by sampling out and back again along two additional altitudes, trying to keep at least one of them within the boundary layer to obtain information on vertical gradients in composition. During a typical 8-h flight, the DC-8 could sample along two jetways (or Yellow Sea) in between three stereoroutes over Olympic Park and Taehwa Research Forest.

To complement the in situ sampling by the DC-8, the NASA King Air flew at higher altitudes (typically between 8.5 and 9 km) in two modes. The first mode was to fly along the jetways being sampled by the DC-8. Given the shorter endurance (approximately 4 h) and slower flight speed of the aircraft, two sorties would be flown going out along one jetway and back along another or out and back along the same jetway to cover the same flight lines as the DC-8. A second mode was to fly a raster pattern to map trace gas distributions over a specific region. Given the swath width of the GeoTASO spectrometer, a 6-km spacing of the parallel flight legs allowed for full coverage of the area overflown. Figure 3 shows an example of a raster flown over the Seoul Metropolitan Area. Rasters were flown over portions of the Seoul area on 11 occasions, twice over Busan and twice over point sources along the northwest coast. The raster flights over Seoul and Busan repeatedly crossed jetways and airspace control boundaries, requiring special permission from air traffic authorities for operations at an agreed constant altitude.

The Hanseo King Air was able to serve a unique role as it was able to use its smaller size to perform in situ sampling both along and off the jetways when needed. Flight lines in Figure 3 show that its focus was weighted toward the jetway on the western side of the peninsula, the Seoul Metropolitan Area, and point sources along the northwest and southeast coasts. It also routinely executed the same descent path over Olympic Park as the DC-8. It was the only aircraft to fly over the portions of Seoul north of the Han River (see expanded view in Figure 3). This required additional preparation and paperwork as well as landing the aircraft for inspection at Gimpo International Airport before flying north of the river. While this was only done once near the end of the study, it was useful to demonstrate that future flights could be planned to collect data for this important region.

In addition to the special cases described above, flights off the jetways by all of the aircraft were only conducted twice and by special request on Sundays when military flights were minimal. These two occasions were used to sample extensive point source emissions along the northwest coast where a large number of power plants, chemical refineries, and industrial facilities are located. The first occasion was under easterly flow, allowing sampling of plumes from these facilities at various distances downwind over the Yellow Sea. On the second occasion, wind conditions were less favorable, but direct sampling of these facilities was accomplished by flying circular patterns around each point source.

In total, the DC-8 flew 20 sorties, the NASA King Air flew 30 sorties, and the Hanseo King Air flew 33 sorties.

## 3. Air quality conditions in Korea during the study

**Figure 4.**
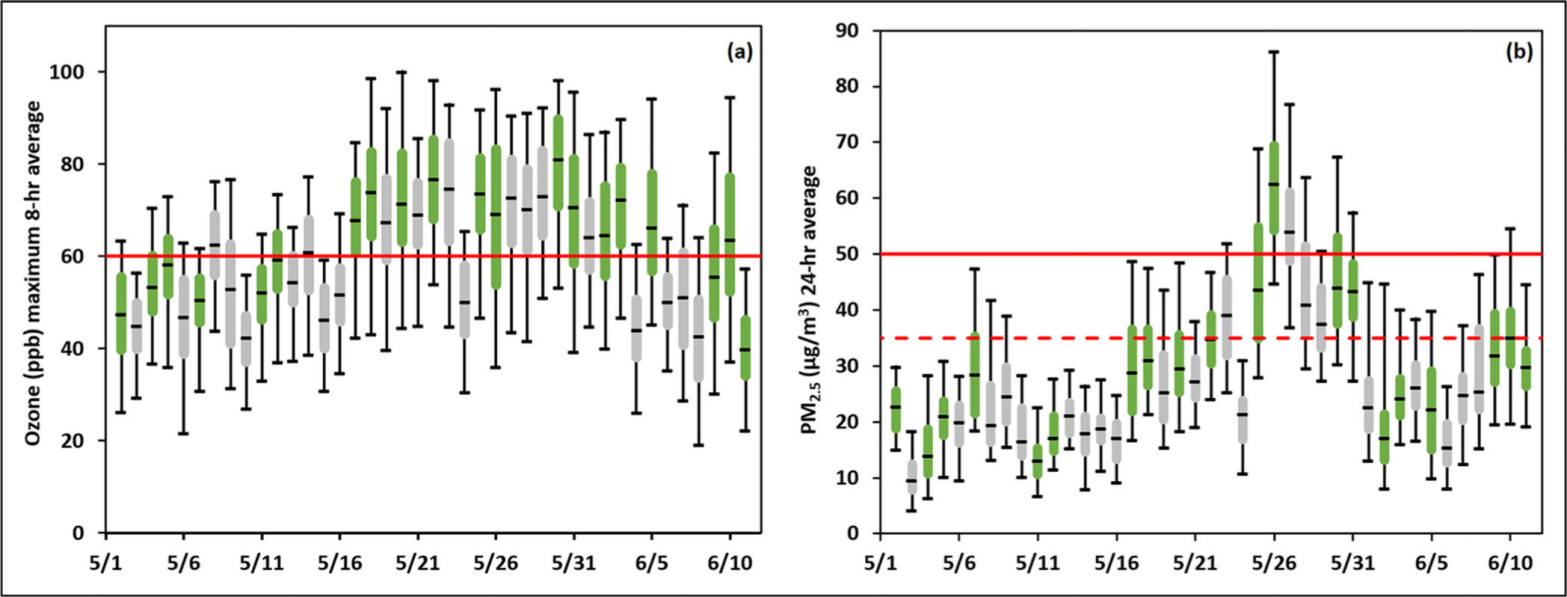
Daily statistics for (a) ozone and (b) PM_2.5_ observed across the AirKorea monitoring network during the Korea-United States Air Quality field study. Box whisker plots indicate the median, interquartile range, and 5th and 95th percentiles. Flight days are shown in green. Red lines indicate the air quality standards in place at the time of the study. The dashed line signifies the more recent tightening of standards for PM_2.5_. DOI: https://doi.org/10.1525/elementa.2020.00163.f4

The timeframe of the KORUS-AQ study was selected to occur during the pre-monsoon season when there would be potential for both ozone and fine particulate pollution episodes (Ghim and Chang, 2000; Lee et al., 2009). Based on climatology, this period was also expected to emphasize the role of local emission sources under the control of South Korean policy makers. Figure 4 provides a statistical summary of air quality conditions during KORUS-AQ based on data collected from the AirKorea monitoring network for ozone (O_3_) and fine particulate matter having a diameter of less than 2.5 μm (PM_2.5_). While ozone exceeded the 60 ppb daily 8-h standard somewhere in the network on most days, the preponderance of high ozone values occurred during the latter half of May and into early June. Overall, 47% of the ozone data collected across the network during the study period exceeded the 8-h standard. In addition, 15% of the data exceeded the 1-h standard of 100 ppb (not shown). Flight days, highlighted in green, show that the overall trend in surface ozone during the study period is also well sampled for conditions aloft. The trend for surface PM_2.5_ is also well captured by the flights, but it is quite different, with a much narrower timeframe for peak values during the last week of May. Values exceeding the daily-average standard of 50 μg/m^3^ were recorded for only a few days. Overall, 8% of the PM_2.5_ data collected across the network during the study period exceeded the daily standard; however, under the recent reduction of the daily standard to 35 μg/m^3^ (dotted line) in early 2018, exceedances increase to 26%.

The meteorological conditions associated with these timelines and their relevance to air quality conditions are discussed in detail by Peterson et al. (2019). Peterson et al. divide the KORUS-AQ study into four distinct periods:

May 1–16: Dynamic meteorology and complex aerosol vertical profiles

May 17–22: Stagnant conditions under a persistent anticyclone

May 25–31: Dynamic meteorology, low-level transport, and haze development

June 1–7: Blocking pattern

These periods played a particularly important role in the analysis and interpretation of the timeline for PM_2.5_, especially given the role of local versus transboundary influences between the stagnant and low-level transport/haze periods in the latter part of May. By contrast, the stronger role of local emissions on the photochemical production of ozone resulted in less dramatic shifts in response to synoptic meteorology. Instead, strong ozone gradients were more common on the shorter timescales associated with sea breeze fronts. This phenomenon is discussed in detail by Peterson et al. (2019).

## 4. KORUS-AQ findings related to controlling factors for air quality

Initial KORUS-AQ findings were published 1 year after the study in a Rapid Science Synthesis Report (RSSR) available in both English and Korean on the KORUS-AQ website (NIER, NASA, 2017). Some findings of that report are still valid, but they were not peer reviewed, and significant new information has come to light through the detailed modeling and analysis of KORUS-AQ observations. An updated and more complete set of peer-reviewed findings published in this special issue and other journals are summarized here. These updated findings provide the basis for the Final Science Synthesis Report (FSSR).

### 4.1. Fine particle pollution

South Korea suffers from both long-term and episodic problems with fine particle pollution. That does not discount the progress that has been made in reducing PM over the last two decades (Kim and Lee, 2018). In March 2018, South Korea tightened standards for daily average PM_2.5_ from 50 to 35 μg/m^3^, consistent with that of other developed countries, as part of their Comprehensive Plan on Fine Dust Management. Monitoring of PM_2.5_ was first implemented in the AirKorea network in 2015 and has continued to expand, providing more complete information on the extent of affected areas. As South Korea continues to work and develop plans to further reduce PM pollution, the ground-based and airborne observations of fine particle properties and composition during KORUS-AQ provide a valuable snapshot with sufficient detail for understanding the factors controlling PM pollution episodes during the study period.

Early research findings focused on modeling and analysis to determine the balance of local and transboundary contributions to PM_2.5_ variability. Kim et al. (2018) contributed an in-depth analysis of data collected by a high-resolution time-of-flight aerosol mass spectrometer (HR-ToF-AMS) at the Korea Institute of Science and Technology (KIST) in Seoul. This analysis revealed the dominance of secondary aerosol (76%) throughout the study period and noted a shift in particle composition between the stagnant and low-level transport periods. The stagnant period was found to be dominated by secondary organic aerosol (SOA), whereas the peak in particulate matter during the low-level transport/haze period was driven by an increase in the inorganic aerosol components due to a combination of transport and meteorology favorable to local haze formation. Nault et al. (2018) evaluated observations of SOA from another HR-ToF-AMS onboard the NASA DC-8 and determined that photochemical processing of local emissions dominated the SOA budget over Seoul during the study. This was supported by several lines of evidence that included correlation of SOA with other photochemically derived trace gases (e.g., O_3_, CH_2_O, and peroxy acyl nitrates), background analysis of CO and organic aerosol over the Yellow Sea upwind of Seoul, use of an oxidation flow reactor showing air over Seoul to have an SOA formation potential 3.5 times greater than upwind over the Yellow Sea, and simple box-model calculations to estimate precursor contributions. Model results indicated that short-lived aromatic compounds played a dominant role as SOA precursors, accounting for about one third of observed SOA.

A modeling study by Choi et al. (2019) employed the GEOS-Chem model with high-resolution nesting over Northeast Asia to estimate the balance between local and transboundary contributions to PM_2.5_ during the KORUS-AQ period. The model showed that local contributions ranged from 26% to 57% with a strong dependence on meteorology. While transboundary influence from China peaked at 68% during the pollution extremes observed during May 25–28 (see Figure 4), this was also the period during which the model exhibited the largest deficit between predicted and observed PM_2.5_ with differences of up to 30–40 μg/m^3^. This difference suggested that additional processes either unrepresented or poorly represented by current models were playing an important role in boosting the PM_2.5_ abundance during this period.

One hypothesis raised by Eck et al. (2020) pointed to increased rates of gas-to-particle conversion in the high relative humidity and enhanced cloudiness associated with the meteorological conditions responsible for trans-boundary transport. Evidence was based on combining AERONET remote sensing of AOD and column-integrated aerosol properties with DC-8 in situ aerosol and water vapor measurements as well as ground-based PM_2.5_ from AirKorea monitoring sites. General increases in AOD associated with larger retrieved fine mode particle sizes detected by AERONET during cloudy periods provided strong evidence of the humidification of aerosols and was corroborated by DC-8 in situ observations of dry and ambient aerosol optical properties. Under these conditions, particle volume size distributions were an order of magnitude greater than those observed under clear conditions and were dominated by fine mode particles (>90%), demonstrating the greater aerosol water content available for heterogeneous processing during cloudy periods. Evidence for more rapid gas-to-particle conversion came from a comparison of PM_2.5_ for AirKorea sites in central Seoul with those from coastal areas to the west of Seoul in Incheon. The largest differences in PM_2.5_ occurred during the period of transboundary transport with values in central Seoul being greater than coastal values by as much as 45% (25–26 May), suggesting that the emissions of gas-phase pollutants that are concentrated in Seoul were being heterogeneously processed to enhance PM_2.5_ over the city compared to its surroundings. A longer-term evaluation of AERONET observations at Yonsei University over an 8-year period demonstrated that the increases in AOD and fine mode fraction size distributions were a regular occurrence during May–June, suggesting that the KORUS-AQ period was not unique.

**Figure 5.**
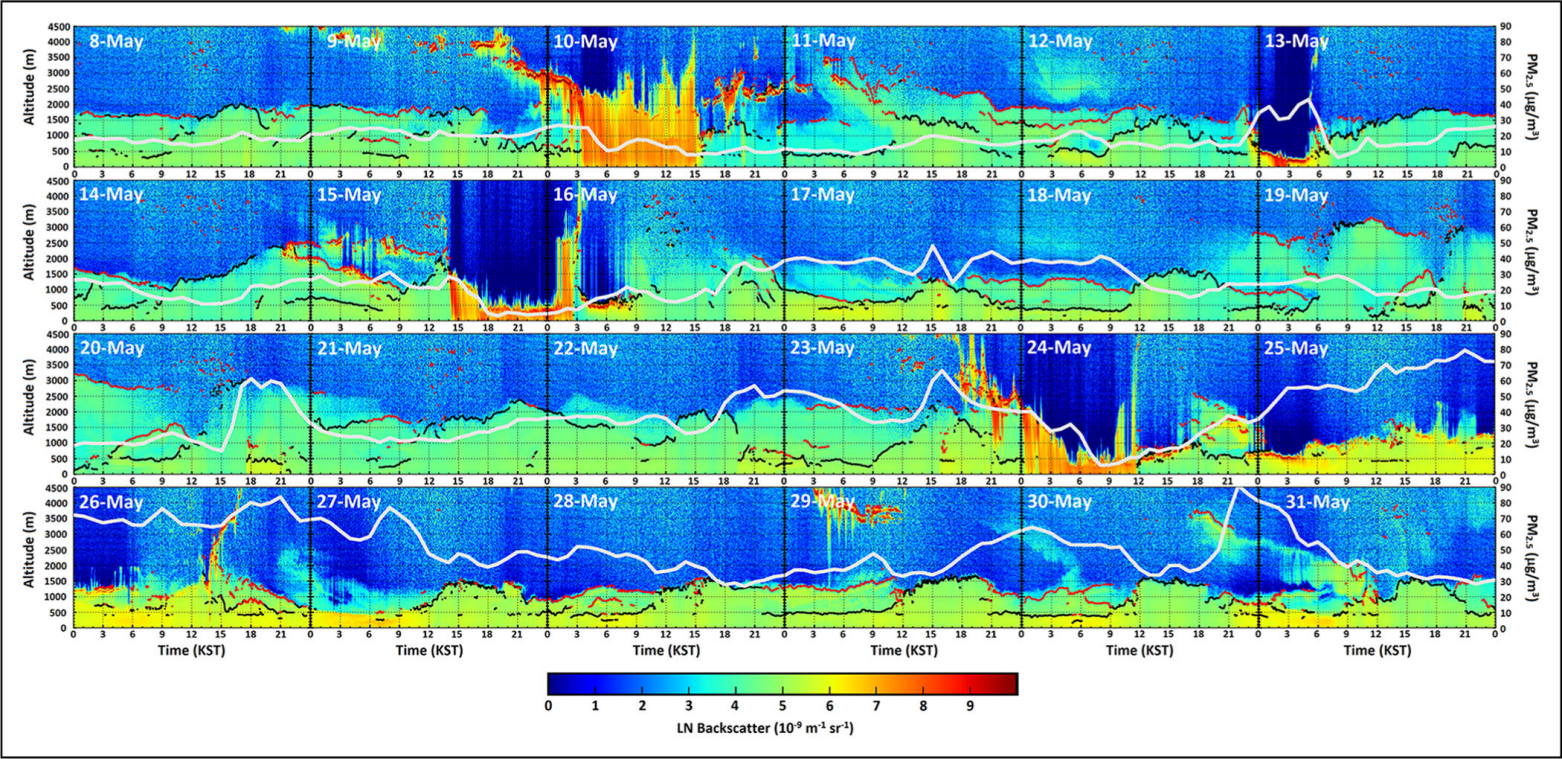
Ceilometer (CL51) normalized backscatter images of the lower atmosphere (0–4,500 m) over Olympic Park from May 8 to 31, 2016. Black and red lines indicate backscatter gradients associated with mixed layer and residual layer heights diagnosed from the CL51 BL-View 1.0 graphical interface with statistical filter applied (Knepp et al., 2017). The white line shows variability in hourly-average PM_2.5_ (0–90 μg/m^3^) for AirKorea monitors in Seoul. DOI: https://doi.org/10.1525/elementa.2020.00163.f5

Building on the work of Eck et al. (2020), Jordan et al. (2020) performed a detailed analysis of the timeline of PM_2.5_ observations across the full AirKorea network and the comprehensive gas-phase precursor and aerosol observations from Olympic Park and other sites in Seoul, including DC-8 profiles over the city. Ceilometer data from Olympic Park and hourly average PM_2.5_ across AirKorea monitors in Seoul from May 8 to 31 are shown in Figure 5 and provide a visual framework for further understanding the meteorological factors at play by bringing boundary layer mixing into focus as an important contributor by trapping pollutants. A common feature in the time series is the association of elevated PM_2.5_ with periods of higher backscatter confined to shallower layers in the ceilometer observations. This is most prominent during the trans-boundary transport period following the clean out of PM_2.5_ by the precipitation and frontal passage on 24 May. Another clean out of PM_2.5_ is seen for the frontal passage late on 15 May. There are also short and abrupt changes in PM_2.5_ (e.g., afternoon of 20 May) associated with local effects such as sea breeze fronts (see Peterson et al., 2019, for more details). The lack of ventilation due to weaker vertical mixing under the cloudiness of the transport/haze period places greater emphasis on local sources than might be expected. Halliday et al. (2019) developed a chemical diagnostic based on short-term ΔCO/ΔCO_2_ slopes from the DC-8 observations for which enhanced slopes (i.e., lower combustion efficiencies) were an indicator of Chinese influence. Using this diagnostic, Jordan et al. (2020) show clear evidence for transboundary influence from China during the transport/haze pollution episode, but the diagnostic was enhanced to a much greater degree in the lower free troposphere compared to the boundary layer. This suggests that the shallow boundary layer both traps local pollution and reduces the potential for entrainment of air from upwind sources during this period.

Jordan et al. (2020) also looked more deeply into the chemical response to the meteorological transition between the stagnant and transport/haze periods. The primary indication of a change in chemistry was the shift in aerosol composition from organically dominated during the stagnant period to inorganically dominated during the subsequent transport/haze period. This change was observed across sites in Seoul as well as all of the supersites distributed across the country (Table 3). DC-8 observations revealed that the shift in composition was accompanied by increased aerosol hygroscopicity, and thermodynamic calculations indicated increases in aerosol liquid water (ALW) by a factor of three. This evidence provided additional support for the hypothesis of Eck et al. (2020) regarding enhanced heterogeneous chemistry under the humid conditions of the transport/haze period.

Trace gas observations provided evidence of enhanced nitrate formation during the transport/haze period. Nitrogen oxide (NO) emissions in Seoul routinely result in the removal of near-surface ozone at night with ozone reaching minima of only a few ppb. During the peak of the PM_2.5_ episode, this removal did not occur, and ozone remained in the 20–40 ppb range throughout the night. This indicated deeper vertical mixing at night, allowing nocturnal emissions of nitrogen oxides to be more thoroughly converted to nitrate at rates calculated to be roughly three times faster during the transport/haze period. This is consistent with the impact of cloudiness on vertical mixing, which reduces vertical mixing during the day but enhances it during the night through trapping of thermal radiation.

The body of work evaluating the KORUS-AQ observations demonstrates that reducing local emissions will benefit fine particle pollution under all conditions. For example, reductions of aromatic VOC emissions will benefit organic-dominated conditions such as those observed during the stagnant period and reductions of NO_*x*_ emissions will benefit inorganic-dominated conditions similar to the transport/haze period. These results also highlight the need for deeper scrutiny of aerosol composition in global and regional models. While synoptic-scale transport is well represented, other responses related to vertical mixing and humidity and their impact on heterogeneous processing and nocturnal chemistry need dedicated attention before large-scale models can properly attribute the sources driving fine particle pollution.

### 4.2. Ozone pollution

**Figure 6.**
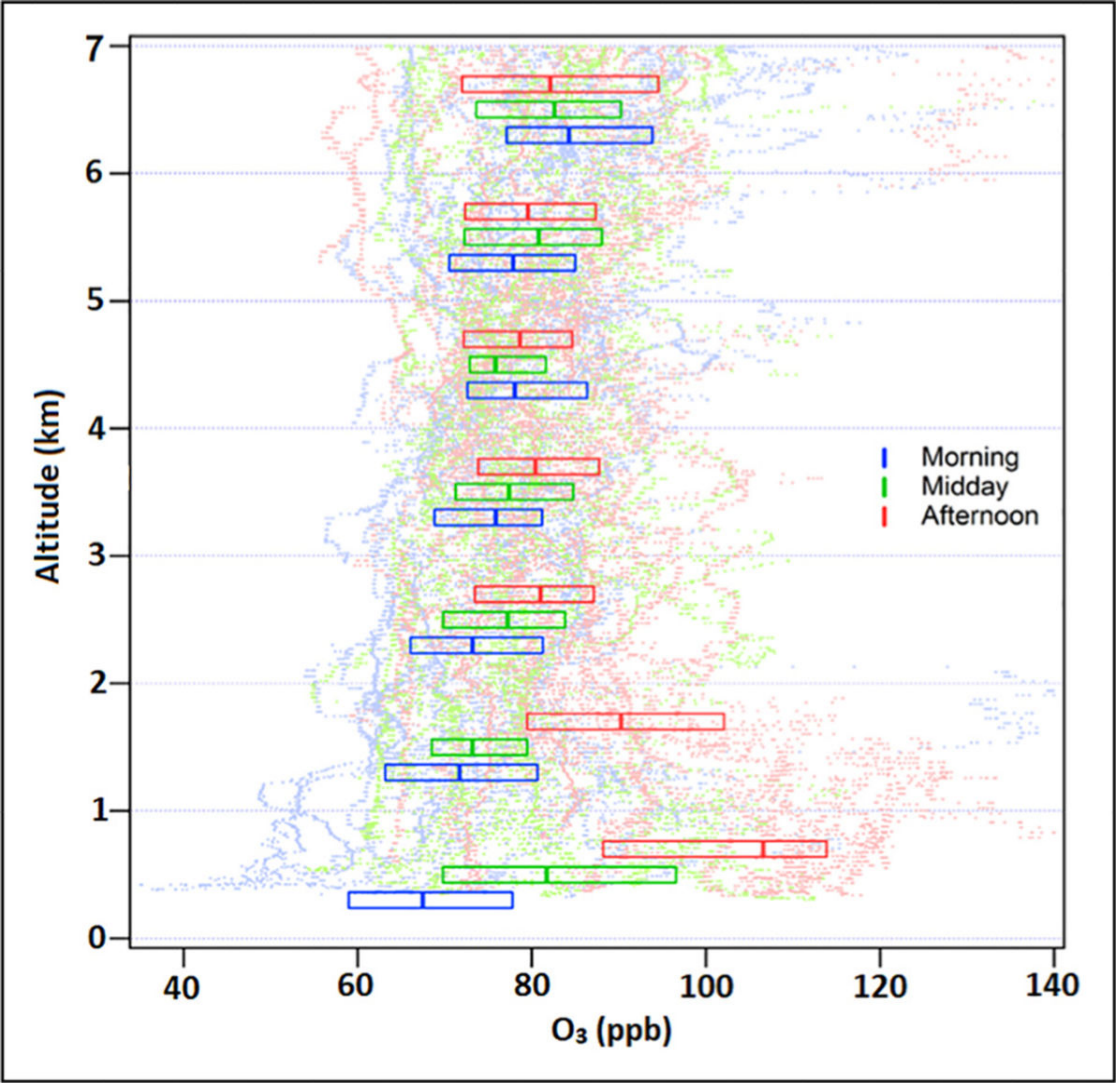
Vertical distribution of ozone observed by the DC-8 during 52 profiles conducted over the Seoul Metropolitan Area east of the Taehwa Research Forest site. Boxes showing median and inner quartile values for 1 km increments of altitude are plotted over the individual measurements separated into morning, midday, and afternoon observations. DOI: https://doi.org/10.1525/elementa.2020.00163.f6

Ozone pollution in South Korea has undergone a slow, seemingly inexorable rise over the past three decades (Kim and Lee, 2018). Just as with fine particle pollution, there are elements of local and transboundary influences to consider. Figure 6 shows the diurnal statistics of ozone with altitude for the DC-8 spirals conducted east of Seoul near the Taehwa Research Forest supersite. A distinct diurnal pattern is observed in the lowest kilometer with median ozone values ranging from 68 ppb in the morning to 107 ppb in the afternoon. This wide range is related to both the overnight and early morning titration of ozone and the afternoon culmination of photochemical ozone production. Moving up to 1–2 km, median ozone values cover a smaller range (72–90 ppb) as the morning effects of titration by surface NO_*x*_ emissions are reduced, and boundary layer depths in the afternoon do not always reach 2 km. The effect of photochemistry in this layer is still quite large with ozone changing by almost 20 ppb between morning and afternoon. Perhaps most striking are the layers between 2 and 4 km where median ozone values are in the vicinity of 75–80 ppb and almost all values exceed the 60 ppb 8-h average ozone standard for South Korea. This demonstrates that a substantial background ozone problem exists in addition to the local ozone production near the surface such that entrainment of air from the lower free troposphere alone is sufficient to violate the ozone standard. Whether the more modest ozone production rates (5–8 ppb) in the lower troposphere (2–4 km) is locally or regionally driven is immaterial to the idea that ozone can only be improved through a combination of measures taken both locally by Korea and regionally by neighboring countries.

Investigation of ozone pollution was conducted using both a 0-D box model (Schroeder et al., 2020) and a 3-D global chemistry transport model (Oak et al., 2019). These studies were complementary given the relative strengths of each approach. Box models have the distinct advantage of assessing chemical production rates directly from observed conditions; thus, they are ideal for examining the detailed sensitivity of ozone production to precursors. 3-D global chemistry transport models include full treatment of emissions, chemistry, transport, and physical processes; thus, they allow assessment of ozone abundances and their response to changes in emissions. While not detailed here, each study provided careful scrutiny and evaluation of model performance to provide confidence in their results.

Schroeder et al. (2020) used a 0-D box model to calculate net ozone production rates for the Seoul Metropolitan Area based on the comprehensive DC-8 observations of precursors, photolysis frequencies, and photochemical products. By evaluating ozone production across the strong gradient in nitrogen oxides between Olympic Park and Taehwa Research Forest, Schroeder et al. confirmed the NO_*x*_-saturated (VOC-limited) conditions that prevail in the Seoul Metropolitan Area. This assessment is corroborated by others (Kim et al., 2018; Kim et al., 2020a). Sensitivity calculations were then conducted by eliminating different types of VOCs from the model and assessing changes in ozone production rates. By far, the largest response was found for C7+ aromatic compounds (e.g., toluene and xylenes) which accounted for a 32% reduction in mean ozone production when removed from the model. Significant response was also found for alkenes (14%), not including isoprene (15%) which was evaluated separately due to its biogenic source. These compounds far outweighed the impact of other VOC classes; thus, they represent the most important targets for ozone reduction strategies.

Recognizing that the regional extent of ozone production in the Seoul Metropolitan Area is still tied to NO*x* emissions, Schroeder et al. also calculated responses in ozone production to reduced levels of NO*x*. Given that NO*x*-saturated conditions suppress the cycling of peroxy radicals responsible for ozone production, these calculations demonstrated two effects. First, reductions in NO*x* would lead to greater rates of ozone production, and second, the increase in chemical cycling under reduced NO*x* conditions would shorten NO*x* lifetimes and reduce the spatial extent of NO*x* more rapidly than the simple rate of reduction in emissions. The interaction of these two effects, however, requires the use of a 3-D chemical transport model to fully quantify impacts on the NO*x* distribution and resulting ozone abundances.

Oak et al. (2019) used the GEOS-Chem chemical transport model to examine ozone production efficiency. Three key adjustments to the model included updating the model with detailed aromatic chemistry, scaling the diurnal variation in planetary boundary layer (PBL) height based on lidar observations, and increasing NO_*x*_ emissions by 50%. These adjustments brought the model into better agreement with observations and also compared well with the observation-based box model results of Schroeder et al. (2020).With the larger regional perspective provided by the GEOS-Chem model, Oak et al. were able to show the gradients in ozone photochemistry between VOC-limited urban/industrial complexes and the greater South Korean peninsula. While the Seoul Metropolitan Area and industrial point sources along the northwest coast stood out, VOC-limited conditions were also present in other coastal urban/industrial regions that included Busan, Pohang, and Ulsan in the southeast, Yeosu in the south, and Gangneung in the east. Sensitivity analyses showed that reducing NO_*x*_ alone by 30% would lead to ozone increases in these urban/industrial zones while benefitting the rest of the peninsula. Another scenario with concurrent reductions in both NO_*x*_ and VOC emission by 30% indicated that ozone would be reduced even further across most of the peninsula while largely avoiding ozone increases in urban/industrial areas.

A detailed analysis of ozone at the Olympic Park supersite (Kim et al., 2020a) complemented the sensitivity studies of Schroeder et al. and Oak et al. by examining high ozone episodes. While meteorology was shown to play a role in determining ozone levels, high ozone was observed across all meteorological regimes and was ultimately tied to conditions of high UV and greater precursor concentrations in the early morning compared to non-episode days. Thus, precursor emission reductions may temper the severity and/or frequency of ozone episode days by limiting peak precursor concentrations.

Olympic Park observations also revealed an interesting but yet unpublished result from J. Gil et al. (private communication) showing that peak nitrous acid (HONO) abundance in the morning could be related to daily maximum ozone in the afternoon. This suggests that the additional source of radicals from HONO photolysis in the early morning could increase the morning rates of ozone formation and contribute substantially to peak ozone values in the afternoon. The presence of elevated HONO has been reported in many polluted urban environments and a complete elucidation of HONO chemistry is still an area of active research (e.g., Zhang et al., 2020).Thus, the role of HONO in ozone formation is still not well represented in models. If the impact of HONO chemistry could be substantiated, this additional contribution to ozone production would render NO_*x*_ emission controls more effective for reducing ozone than indicated by conventional model predictions.

Ozone and fine particle pollution share overlap in their sensitivity to NO_*x*_ and VOC emissions, with aromatic VOCs being of particular importance to both types of pollution. This encourages joint mitigation strategies for addressing ozone and PM_2.5_ together. However, unlike fine particle pollution, ozone mitigation calls for additional consideration of the relative rates of NO_*x*_ and VOC reductions. The VOC-limited conditions present in the most populated areas call for initial reductions to be more aggressive for VOCs. This will minimize the potential for degraded ozone conditions in densely populated areas that could occur before reductions of 30% or more in both NO_*x*_ and VOCs can be achieved.

### 4.3. Emissions

Effective targeting of NO_*x*_ and VOC emission reductions requires detailed knowledge of the various source types and their distributions. KORUS-AQ observations enabled assessment of the magnitude of emissions as well as the apportionment of emissions across various sources. The updated emission inventories provided in support of KORUS-AQ contributed to improving model representation of conditions observed during the study. Detailed analysis of VOC observations provided information on emission sectors worthy of targeting for VOC emissions reductions. Observations also enabled top-down assessment of point source emissions, revealing important underestimations for VOC emissions while confirming emission estimates for NO_*x*_ and SO_2_. Each of these contributions is described in more detail below.

#### 4.3.1. Emission inventories

During each stage of the study, detailed bottom-up emissions were provided to the participating modeling teams. These emissions were provided in a model-ready form (e.g., standard gridding, monthly variation, and speciation) that could be used with minimal additional processing required to fit specific model needs (Woo et al., n.d.). The initial inventory, KORUSv1, was provided for use during the execution of the study and was used in air quality forecast models to support flight planning. KORUSv1 was based on 2010 emissions projected to 2015.

An updated version, KORUSv2.1, was based on updated emissions projected from 2012 for Korea and from 2015 for the rest of Asia. These emissions also included new VOC speciation profiles that provided important details on the chemical composition specific to different sectors of importance, for example, solvent use, mobile sources, residential sources, and industrial processes (Woo et al., n.d.). KORUSv2.1 emissions were used in the post-mission analyses of PM_2.5_ (Choi et al., 2019) and ozone (Oak et al., 2019). The need for additional updates was evident in the work of Oak et al., which included a 50% increase in NO_*x*_ emissions to improve model predictions in comparison to observations. An underestimation of aromatic VOCs was also noted, but the chemical consequences for ozone were small in comparison to NO_*x*_. An additional piece of evidence for larger NO_*x*_ emissions came from the work of Goldberg et al. (2019b), who conducted a top-down analysis using OMI satellite observations and high-resolution WRF-Chem simulations to estimate NO_*x*_ emissions that were 1.37 times greater than the inventory for the Seoul area and more than two times greater near large industrial sources.

A final update to the emissions inventory, KORUSv5, developed in 2019 was able to take advantage of updated inventories that became available in 2018 for Korea (CAPSS 2015), China (MEIC 2016), and Japan (PM2.5 EI 2015). Korean NO_*x*_ emissions in KORUSv5 were also increased by 37% to reflect the findings of the top-down analysis of Goldberg et al. This was supported by the more recent finding that on-road mobile emission factors in Korea should be 30% higher based on determinations from portable emissions measuring systems (PEMS; see Woo et al., n.d.).

KORUSv5 emissions were used to support a multi-model intercomparison for the KORUS-AQ period (Park et al., 2021). The intercomparison showed that the ensemble of model results performed better than the individual models in predicting observed variability of PM_2.5_ and O_3_ and that model performance was generally improved over earlier predictions that used the original KORUSv1 inventory. Diversity in model performance in representing PM_2.5_ composition showed the need for ongoing model development and evaluation.

#### 4.3.2. VOC source apportionment

For VOC emissions, a detailed analysis was conducted by Simpson et al. (2020) on the relative abundance and reactivity of specific compounds as well as their source apportionment. The analysis centered on whole air samples (WAS) collected from the DC-8, which provided information on more than 80 different VOCs and tracers (see Table 4). WAS data were supplemented by concurrent observations from other instruments on the DC-8 including CO, CH_4_, HCN, and oxygenated VOCs. Through spatial segregation and tracer analysis, Simpson et al. isolated specific source signatures of emissions associated with Seoul, Busan, the Daesan petrochemical complex southwest of Seoul, and transboundary transport from China. When considering both the relative abundance and reactivity of individual VOCs, results corroborated the dominant influence of aromatics on local ozone production with important contributions from alkenes and isoprene.

Using positive matrix factorization (PMF), Simpson et al. provided additional details on source apportionment by presenting both four- and five-factor solutions based on the observations collected over Seoul. The first three factors were very similar between the two solutions. Factor 1 isolated biogenic emissions. Isoprene was the only species making a significant contribution (>80%) to this factor in both solutions. Factor 2 was most consistent with long-range transport of emissions from China, exhibiting strong contributions from CO and carbonyl sulfide as well as longer lived tracers, ethane and ethyne. Factor 3 suggested vehicle exhaust as it had the strongest alkene contributions along with light alkanes. In the four-factor solution, solvents and LPG/gas emissions were indicated in the fourth factor. This factor also included light alkanes, but aromatic compounds and heavier alkanes associated with solvents were dominant. The five-factor solution provided the added benefit of differentiating paint from non-paint solvents. This was exhibited in the separation of xylenes and ethylbenzene more expected from paint in one factor, while the other factor indicated toluene-rich emissions along with heavy alkanes indicative of solvents associated with consumer products and printing. The PMF analysis complements the PM and ozone analyses described in Sections 4.1 and 4.2 by identifying the sectors responsible for the largest contributions to ozone and organic aerosol production across the Seoul Metropolitan Area and by providing clear target species to consider for reduction. It is important, however, that specific products responsible for aromatic emissions within the broad categories of paint, consumer products, and printing be further identified and regulated.

#### 4.3.3. Top-down assessment of point source emissions

During KORUS-AQ, direct observation of point source emissions was only an occasional feature of flight plans, but one flight was dedicated specifically to point sources (power plants and industrial facilities) along the northwest coast. Of these sources, the Daesan chemical facility was sampled on several different days. In situ sampling was accomplished by flying in a circular pattern around each point source, varying the size of the circle to capture the emitted plume at two to three downwind distances. Sampling was also accomplished with remote sampling overflights of the GeoTASO instrument.

VOC emissions from the Daesan facility were assessed by three separate groups. Fried et al. (2020) performed the most direct assessment using a mass balance approach to estimate total VOC emissions as well as emissions of CH_2_O and its four main precursor gases (ethene, propene, 1,3-butadiene, and 1-butene). Results indicated top-down emission rates that were much higher than those reported in the bottom-up inventory by factors of 2.9 ± 1.0 for total VOCs and 4.3 ± 1.9 for CH_2_O and its four major precursors. These results were corroborated by a top-down estimate based on CH_2_O column densities remotely sensed by GeoTASO (Kwon et al., 2021) that exceeded the bottom-up inventory by a factor of 4.0 ± 2.3. The third top-down estimate by Cho et al. (n.d.) used a box model to evaluate the build-up in CH_2_O between downwind plumes of the Daesan facility sampled by the DC-8 and Hanseo King Air separated by approximately 4.5 h. This resulted in a value that exceeded the bottom-up inventory by a factor of 2.5 ± 0.4.

Top-down assessments of SO_2_ and NO_*x*_ proved to be more encouraging. General agreement with the bottom-up inventory was found in two studies. Fried et al. (2020) applied the same mass balance approach to emissions of SO_2_ from the Daesan facility and found a ratio of 0.81 ± 0.29 between top-down and bottom-up emissions. This result gave confidence to the application of the mass balance approach to the more complex VOC emissions described above and the finding that they are underestimated by the inventory. An assessment of five additional point sources (Hyundai Steel, Dangjin power plant, Boryeong power plant, Seocheon power plant, and Gunsan industrial complex) was conducted by comparing DC-8 measurements to a Gaussian plume model (Park et al., n.d.). These point sources are monitored directly by CleanSYS monitors that provide real-time stack emissions. The agreement between modeled and measured concentrations was in the range of 0.83–1.26 for NO_*x*_ and 0.74–0.91 for SO_2_. This demonstrates the value of stack monitors for providing accurate point source emissions.

## 5. Role of satellite observations

Satellites offer a valuable perspective for understanding air quality. Broad spatial coverage reveals information on the distribution of pollutant emissions and precursors (e.g., NO_2_, CH_2_O, SO_2_, and CO) as well as secondary pollution related to tropospheric ozone and aerosol optical depth (which is a proxy for particulate matter). Satellites observe these constituents as column-integrated quantities; thus, their interpretation requires careful validation and further relies on integration with in situ observations and models that can synthesize the underlying chemical and dynamical processes governing the observed distributions. Historically, the broad spatial coverage and daily observations from satellites in LEO have enabled global views of emissions and their long-term trends (e.g., Richter et al., 2005; Hilboll et al., 2013; Duncan et al., 2016), their responses to specific policies and events (e.g., Li et al., 2010; Russell et al., 2012; Lelieveld et al., 2015), and their transboundary influences (e.g., Park et al., 2014; Cuesta et al., 2018). From geostationary orbit, GEMS will offer the first opportunity to make hourly measurements of key pollutants and observe changes in their distributions on short temporal scales to provide insight into the daily interactions between emissions, chemistry, and meteorology that determine air quality outcomes. The introduction of diurnally varying information across Asia will contribute to improvements in top-down emissions studies, air quality forecasting through assimilation, evaluation of model chemistry and transport, and identification of unmonitored locations deserving more attention.

**Figure 7.**
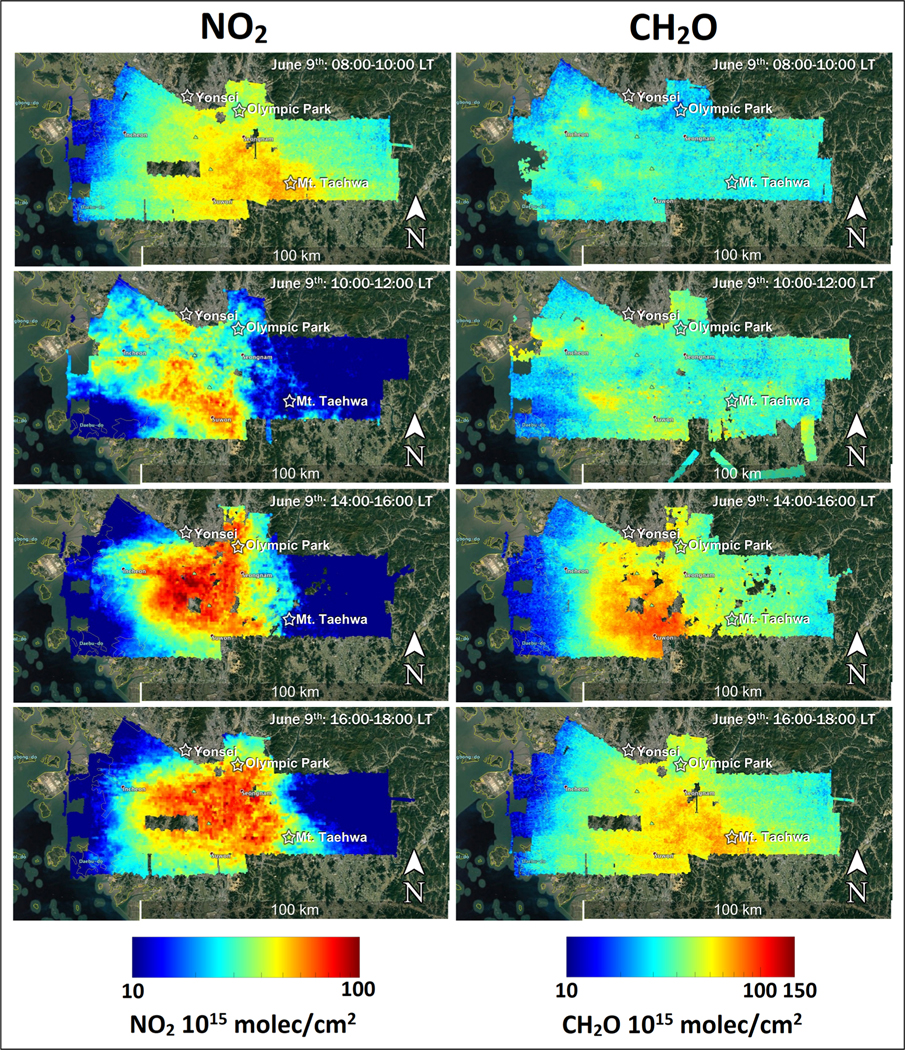
Geostationary Trace gas and Aerosol Sensor Optimization (GeoTASO) mapping of NO_2_ and CH_2_O vertical column densities across the Seoul Metropolitan Area throughout the day on June 9, 2016. DOI: https://doi.org/10.1525/elementa.2020.00163.f7

KORUS-AQ was specifically designed to explore the integration of GEMS into multi-perspective observations to inform air quality as shown in Figure 1. As described earlier, the GeoTASO instrument onboard the NASA King Air provided remote sensing information analogous to what will be available from GEMS. Analyses making use of GeoTASO observations during KORUS-AQ can be found in Chong et al. (2020) and Kwon et al. (2021). Examples of GeoTASO observations for NO_2_ and CH_2_O over the Seoul Metropolitan Area during flights on June 9, 2016, are shown in Figure 7. While the aircraft could only complete four mappings in a single day compared to the 11 hourly images that GEMS will provide, the GeoTASO images are at much higher spatial resolution (250 m × 250 m) compared to that of GEMS (7 km × 8 km). There are several notable features in the distributions. First and most important is that the distributions of NO_2_ and CH_2_O are different, and their intersection leads to variability in chemical rates for ozone and aerosol formation. Given the direct emission of nitrogen oxides, NO_2_ shows areas of concentration early in the day. By contrast, CH_2_O is a chemical product of VOC oxidation, so it exhibits a smoother distribution and is markedly lower in the morning. By afternoon, continued emissions of NO_2_ and light winds on this day move the pollution slowly to the southeast. CH_2_O peaks in the mid afternoon and overlaps significantly with NO_2_, which influences rates of VOC oxidation and promotes reactions that favor CH_2_O formation.

**Figure 8.**
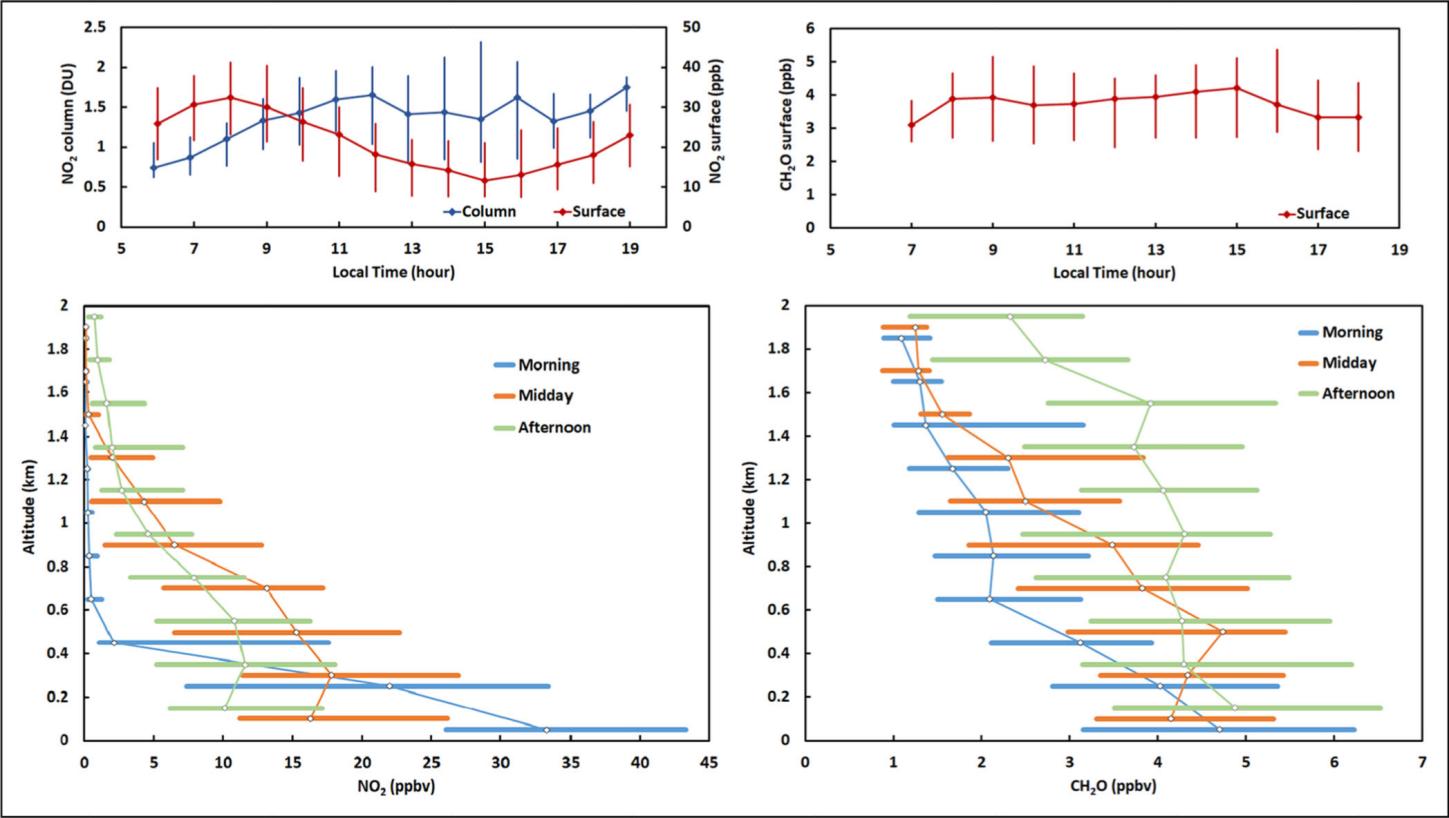
Diurnal statistics for multi-perspective observations of NO_2_ and CH_2_O at Olympic Park. Median and interquartile ranges are shown for hourly in situ surface measurements, hourly Pandora column densities (NO_2_ only), and in situ vertical profiles from the DC-8 for morning, midday, and afternoon overflights. DOI: https://doi.org/10.1525/elementa.2020.00163.f8

The complexity of the relationship between NO_2_ and CH_2_O is further demonstrated by comparing surface in situ observations, remotely sensed columns from a Pandora spectrometer, and detailed profiles from DC-8 descents over the Olympic Park site. Each of these perspectives is shown in Figure 8. Diurnal statistics for NO_2_ exhibit an anticorrelation between surface and column values with surface values peaking in the early morning and the column abundance increasing to greater values in the afternoon. This anticorrelation is related to the vertical redistribution of NO_2_, which shows emissions confined to the lowest 500 m in the morning. Throughout the day, ongoing emissions are mixed to higher altitudes as the boundary layer deepens; however, the persistent emissions and short lifetime for NO_x_ lead to decreases with increasing altitude within the boundary layer at all times of day. CH_2_O exhibits quite different behavior with surface values changing very little throughout the day. Despite earlier publications highlighting Pandora observations of CH_2_O (Herman et al., 2018; Spinei et al., 2018), column observations are not shown due to the discovery of an interference (Spinei et al., 2021). Nevertheless, the DC-8 profiles corroborate the stability in surface CH_2_O values. As a product of VOC oxidation, CH_2_O vertical profiles also show quite different behavior than NO_2_. As the boundary layer deepens and VOC oxidation occurs over a larger volume, CH_2_O is produced at all altitudes and builds up uniformly with column abundances peaking in the afternoon.

These differences in NO_2_ and CH_2_O distributions temporally, spatially, and vertically present challenges to satellite interpretation (Schroeder et al., 2017) and serve as a reminder that the integration of satellite data with multi-perspective observations and models as shown in Figure 1 is the key to providing new insight into emissions and their impacts on air quality from local to regional scales.

Investments in measurements that enhance GEMS validation and serve to connect surface monitoring to GEMS such as the emerging Pandora Asia Network (PAN) are particularly welcomed. Such efforts will extend integrated observations across the many Asian countries in the GEMS field of view and build the community of scientists working to inform and improve air quality across Asia. This vision is captured in the plans for building a Pan-Asia Partnership for Geospatial Air Pollution Information under the leadership of the United Nations Economic and Social Commission for Asia and the Pacific (UNESCAP) and funding from the Korea International Cooperation Agency (KOICA).

## 6. Policy recommendations from KORUS-AQ findings

These summaries of peer-reviewed manuscripts from the KORUS-AQ study serve to highlight the major outcomes that have resulted from ongoing analysis after publication of the initial RSSR in 2017. For more detailed discussion and understanding, readers are referred to the other manuscripts in this Special Issue as well as other referenced papers. Taken as a whole, the body of KORUS-AQ work leads to several overall findings and associated policy recommendations that are restated here and are also provided in a comprehensive FSSR to Korea’s Ministry of Environment for consideration.

It should be emphasized that these recommendations are focused on local actions that can be taken to improve PM_2.5_ and ozone pollution. This does not discount that there are substantial transboundary influences to consider and that regional cooperation is necessary to achieve sustainable improvements in air quality for Korea. Clear steps to reduce local emissions are good faith actions needed to support such regional cooperative efforts.

### (1) Improving both PM_2.5_ and ozone pollution relies on coordinated reductions in both NO_*x*_ and VOCs, specifically higher (C_7_+) aromatic compounds.

Analysis of the KORUS-AQ observations revealed dominant roles for both NO_*x*_ and VOCs in the control of PM_2.5_ and ozone, but the impacts are manifested in different ways.

For PM_2.5_, the observed composition changed from organic-dominated to inorganic-dominated conditions depending on meteorology. Organic aerosol was the dominant fraction of PM_2.5_ during stagnant conditions when clearer skies and abundant sunlight were most favorable for photochemical processing of local VOC emissions. By contrast, inorganic aerosol dominated under transport/haze conditions with evidence that transboundary pollution influence was exacerbated by enhanced gas-to-particle conversion of local emissions under the humid conditions. The role of nitrate aerosol driven by local NO_*x*_ emissions enabled a positive feedback by enhancing ALW content, which facilitated additional particle formation.

For ozone, NO_*x*_ and VOC emissions work together in a complex fashion with VOC emissions having greater control of the rate of ozone formation and NO_*x*_ controlling the regional extent of ozone formation. Given the large abundance of NO_*x*_, ozone formation in Seoul is limited by VOC availability such that if VOC emissions alone are reduced, ozone formation rates in Seoul would decrease; however, NO_*x*_ reductions are necessary to reduce the regional extent of ozone formation affecting the greater Korean peninsula. Given that the NO_*x*_ abundance in Seoul is so great that it suppresses the catalytic chemical cycle of ozone formation, NO_*x*_ reductions could initially offset the impact of VOC reductions and lead to more ozone in the short term for urban areas. To add to the complexity, the tendency toward faster chemical cycling as NO_*x*_ emissions are reduced would also shorten the lifetime of NO_*x*_ such that its atmospheric abundance should decrease faster than emissions.

Observation-constrained modeling revealed the importance of C_7_+ aromatic compounds (e.g., toluene and xylenes), indicating that this single class of VOCs was responsible for a third of organic aerosol and ozone production in the Seoul Metropolitan Area.

While reductions in NO_*x*_ and VOCs will yield immediate benefits for PM_2.5_, the rate of reduction between NO_*x*_ and VOCs is important for ozone. Rapid reduction of NO_*x*_ relative to VOCs raises the expectation of increases in ozone, especially in urban areas. In the face of such setbacks, it will be important to be patient and confident that continued reductions will lead to success in the long term.

### (2) Improved estimates place better bounds on current emissions, but specific sources for higher (C_7_+) aromatic compounds need to be targeted to enable effective control strategies.

Air quality control strategies can only be effective if emissions are well understood. An important outcome of KORUS-AQ research was the improvement of emission inventories.

An underestimation in NO_*x*_ emissions was first diagnosed through top-down assessments using satellite observations and models. In response, the subsequent bottom-up assessment increased on-road mobile emissions based on better emission factors from PEMS. This improves the understanding of what can be achieved with current NO_*x*_ control strategies.

For VOCs, emission speciation profiles were improved to allocate more mass to reactive VOCs, and model representation of aromatic compounds compared to observations improved as a result. The sources of these compounds, however, remain broadly distributed and are poorly understood in detail. An observation-based source apportionment analysis indicated that solvent use is primarily responsible for the large abundance of higher aromatic compounds in Seoul, with toluene being more associated with non-paint solvents and xylenes being more associated with paint solvents. These two categories, however, can be linked to a large number of potential products. Identifying specific products to target for reduction is an essential remaining task.

### (3) Large underestimates of VOC emissions from industrial point sources warrant continued scrutiny and verification.

KORUS-AQ made several visits to sample emissions from point sources along the northwest coast of the Korean peninsula. The top-down assessments of NO_*x*_ and SO_2_ emissions agreed well with CAPSS emissions given the supporting observations from the continuous emissions monitoring system. By contrast, multiple top-down assessments based on observations at the Daesan chemical facility showed VOC emissions to be underestimated by a factor of 2.5–4. Given the complex chemical mixtures containing multiple hazardous air pollutants and their oxidation products, underestimation of these emissions carries additional risk to workers and local communities. VOC emissions are different than stack emissions of NO_*x*_ and SO_2_. They occur mainly as fugitive emissions that come from a multitude of facility components and activities such as storage tanks, transport pipelines throughout the complex and to/from the nearby shipping port, petroleum production and handling, as well as combustion and flaring operations. Thus, they cannot be easily measured from a single point in the facility. The recent establishment of the Seosan supersite on the Taean peninsula is a positive step given its proximity to Daesan and other points sources along the northwest coast. However, the discrepancies at Daesan raise questions about whether similar underestimates exist at other facilities, both large and small, across Korea. A top-down airborne survey of point sources may be the most efficient way to determine the extent of this underestimate.

### (4) Model simulations of air quality require a hierarchy of models to obtain the best representation and understanding of uncertainties to support decision making.

KORUS-AQ assembled a team of air quality modeling groups to support the planning, execution, and research stages of the study. These groups employed a hierarchy of models covering regional-to-global domains with various resolutions and various complexity in their treatments of ozone and PM formation. Models play an important role in evaluating emission estimates, providing the translation from emission inventories to ambient concentrations to be compared with observations. The average of several different models, each with differing strengths and weaknesses, was generally better at matching observations than any individual model. This illustrates the value of having multiple models for determining the drivers of air quality and placing uncertainty bounds on quantitative assessments. Ongoing model development, leading to more accurate simulations of ozone and secondary aerosol formation in individual models, is needed to guide policies on emission controls and improve air quality forecasting.

## 7. A way forward

### 7.1. Monitoring

With the combination of the extensive AirKorea monitoring network and hourly monitoring from GEMS as well as GOCI-II, Korea will have unprecedented information to support continuous air quality monitoring of Korea and the larger regional impacts across Asia. This information will support improved forecasting, top-down emissions estimates, assessment of emission controls, and fundamental understanding of the factors driving air quality. Nevertheless, there are a few small additional investments that will benefit the interpretation of information from monitoring observations.

Given the ongoing need to understand local versus transboundary influences, the following measurements colocated at a single site in Seoul would extend the findings of KORUS-AQ and determine their relevance to understanding the drivers of PM_2.5_ throughout the year. High-quality measurements of CO and CO_2_ would provide continuous information on the strength of transboundary influence based on the large difference in combustion efficiency in Korea as compared to China. Continuous research-grade observations of aerosol composition would provide the details needed to better understand changes in the local rate of secondary aerosol production. Additional routine observations of humidified and dry aerosol scattering would allow for calculation of ALW to better understand the coupled chemical and meteorological processes driving haze events. Also, high-quality ammonia measurements would enable model assessments to be fully constrained in the evaluation of inorganic aerosol formation, aerosol acidity, and related aerosol reaction pathways. Finally, to provide increased attention to the role of mixed layer dynamics on PM_2.5_ abundance, a real-time mixing height data product from the currently operating ceilometer network should be developed to provide continuous information on the diurnal cycle of mixing and ventilation of near-surface pollution.

### 7.2. Emission inventories

Bottom-up emissions play an important role in setting up air quality management policies. Korea has made an effort in developing and refining bottom-up emissions for the last two decades. KORUS-AQ analysis provided a critical review of CAPSS, Korea bottom-up emissions, and Chinese emissions by comparing modeling results with field measurements and developing new sets of emission inventories (named as KORUS emissions) primarily for air quality modeling.

Because of differences in development and purposes, KORUS emissions may not be directly comparable to CAPSS. Discrepancies among multiple air quality modeling results also hamper full validation of KORUS-AQ emissions even for modeling purposes. However, the major assessments of CAPSS emissions that led to the development of KORUS emissions should be noted for future development of CAPSS. This refers specifically to the underestimations of mobile source NO_*x*_ and point source VOCs emissions deduced through top-down methods. With continuous GEMS observations and ongoing changes in emissions due to control policies, top-down assessments will continue to be critical to the scrutiny of bottom-up emissions.

### 7.3. Modeling

A number of state-of-the-science air quality models (WRF-Chem, CMAQ, CAMx, GEOS-Chem) are being used to study ozone and PM pollution in Korea and should continue to be supported for further development and evaluation. Ongoing model improvements, based on improved scientific understanding of chemistry and the complex meteorology affecting Seoul and the Korean peninsula, are needed to improve air quality forecasts. The continued close collaboration that began during KORUS-AQ between the groups developing emission inventories and models will lead to improved emissions and models. With greater confidence in air quality forecasts, real-time policy measures such as traffic restrictions based on air quality forecasts might be considered.

### 7.4. The next phase of US-KOREA cooperation

In December 2018, NIER and NASA signed a Memorandum of Understanding concerning “Cooperation in Pollution Studies, Calibration, and Validation.” Since that time, the GEMS satellite launched in February 2020 and the impending launch of TEMPO in 2022 will bring the air quality satellite constellation closer to fruition.

This agreement demonstrates the commitment to continued cooperation between the United States and Korea to the calibration and validation of GEMS and TEMPO observations and more importantly to the interpretation of the information from these satellites to inform air quality forecasts, improve understanding, and provide value to decision making.

The recent development of the Pandora Asia Network has also been critical to expanding collaboration across Asia and globally through membership in the Pandora Global Network. This effort not only brings scientists together but, under the sponsorship of KOICA and UNES-CAP, brings decision makers into the discussion of local and shared impacts to air quality across Asia.

Continued fieldwork will be necessary with current plans already underway for remote sensing flights of the GEO-CAPE Airborne Simulator (GCAS) instrument for GEMS validation. GCAS is similar to the GeoTASO instrument flown on the NAS King Air for KORUS-AQ. Over the longer term, Korean and U.S. scientists will continue to explore and discuss ideas for field study collaborations to evaluate the changing landscape of emissions and the resulting changes in air quality as we work to achieve our respective national goals.

## Figures and Tables

**Table 1.** Instrumentation at the Olympic Park supersite (Lat/Lon: 37.5216/127.1242). DOI: https://doi.org/10.1525/elementa.2020.00163.t1

Investigator, Institution	Instrument Name/Technique	Species/Parameters Measured
Trace gas measurements		

Seogu Cho, Seoul Institute of Health and Environment	Ecotech gas sensors, AeroLaser 4021, Varian GC450, Met One weather sensors	O_3_, NO, NO_2_, NOx, CO, SO_2_, CH_2_O, volatile organic compounds (VOCs), Meteorology: Temperature (T), relative humidity (RH), wind direction (WD), wind speed (WS), solar and UV radiation
Jinsang Jung, Korea Research Institute of Standards and Science (KRISS)	KENTEK gas analyzers	O_3_, NOx, CO, SO_2_
Deug-Soo Kim, Kunsan National University	Teledyne T200U, Thermo Scientific 42i	NO, NO_2_, NOx, NOy
Russell Long and Andrew Whitehill, US Environmental Protection Agency (EPA)	2B Tech 211,Teledyne T200U, Teledyne T500U CAPS, Aerodyne QCL	O_3_, NO, NO_2_, NOx, CH_2_O
Meehye Lee, Korea University	Fluorescence high-performance liquid chromatography, ion chromatography	H_2_O_2_, CH_3_OOH, HONO
Gangwoong Lee, Hankuk Institute of Foreign Studies	Quantum cascade tunable infrared laser differential absorption spectrometer	H_2_O_2_, HONO
Jae Hong Lee, Harim Engineering, Inc.	Teledyne T400, T265, and 430	O_3_
Saewung Kim, University of California, Irvine	Chemical ionization mass spectrometer (CIMS)	Peroxyacetylnitrate (PAN), ClNO_2_, Cl_2_
Seogheon Kim, Yonsei University	Thermal desorption-gas chromatography-mass spectrometry	VOCs
Dongsoo Lee, Yonsei University	Ion chromatography	Acidic gases: HCl, HONO, HNO_3_, H_2_SO_4_
Jinseok Han, Anyang University	Ion chromatography	Base gases: NH3, dimethylamine (DMA), trimethylamine (TMA)

Aerosol measurements		

Minsuk Bae, Mokpo National University	Particle into liquid sampler-total organic carbon analyzer	Water-soluble organic carbon (WSOC)
Seogu Cho, Seoul Institute of Health and Environment	Sunset organic carbon/elemental carbon analyzer, Thermo FH62C14, XRF625, MARGA	Organic carbon/elemental carbon, particulate matter (PM_10_), PM_25_, PM_1_, trace metals, water-soluble ions
Kitai Kang, ART PLUS Co., Ltd.	Scanning mobility particle sizer (SMPS)	Particle size distribution
Sungroul Kim, Soonchunhyang University	Aethalometer AE-33, AE-51	Black carbon
Jae Hong Lee, Harim Engineering, Inc.	Met One BC1050, Teledyne T640	Elemental carbon, organic carbon, PM_10_, PM_2.5_
Jeonghoon Lee, Korea University of Technology and Education	Thermo MAAP 5012, Brechtel TAP 2901, PTI	Black carbon; absorption at 467, 528, and 652 nm; UV absorption
Chul-Un Ro, Inha University	Scanning electron microscopy/energy dispersive X- ray analysis	Particle imaging
Hye Jung Shin, National Institute of Environmental Research	High-resolution time-of-flight aerosol mass spectrometer	PM_1_ ionic composition
Seong Soo Yum, Yonsei University	Humidified Tandem Differential Mobility Analyzer (HTDMA), TSI CPC3776, TSI CPC3010, DMT CCNC	Dry diameter, kappa, condensation nuclei (3 nm and 10 nm cutoff), cloud condensation nuclei (CCN)

Remote sensing measurements and soundings		

James Szykman, US EPA	Vaisala CL51	Mixed layer height
	iMet-1-RSB Radiosondes, DMT/EN-SCI electrochemical concentration cell (ECC) ozonesondes	O_3_, T, P, RH, WS, WD soundings

**Table 2.** Instrumentation at the Taehwa Research Forest supersite (Lat/Lon: 37.3123/127.3105). DOI: https://doi.org/10.1525/elementa.2020.00163.t2

Investigator, Institution	Instrument Name/Technique	Species/Parameters Measured
Trace gas measurements		

Scott Herndon, Aerodyne	Aerodyne tunable infrared laser direct absorption spectroscopy mini spectrometer	CH_2_O
Russell Long, US Environmental Protection Agency (EPA)	2B Tech 211, Aeroqual 500, Teledyne T500U CAPS	O_3_, NO, NO_2_, NOx
Saewung Kim, University of California, Irvine	Thermo 42i, LGR cavity ringdown spectrometer (CRDS), chemical ionization mass spectrometer, Proton transfer reaction-time of flight-mass spectrometer, CRM-chemical ionization mass spectrometer	NO, NO_2_, ClNO_2_, Cl_2_, volatile organic compounds (VOCs), OH reactivity
		VOCs
Meehye Lee, Korea University	Luminol-gas chromatography (GC), gas chromatograph–flame ionization detector	PAN, VOCs
Youngjae Lee, NIER		O_3_, CO, SO_2_, NOx, CO_2_, H_2_O, Meteorology: Temperature (T), wind speed (WS), wind direction (WD)
Thomas McGee, NASA Goddard Space Flight Center (GSFC)	Thermo 42i, Lufft WS501	O_3_, Meteorology: T, P, RH, WS, WD, solar radiation

Aerosol measurements		

Kitai Kang, ART PLUS Co., Ltd.	Scanning mobility particle sizer (SMPS)	Particle size distribution
Youngjae Lee, NIER		Organic carbon, elemental carbon

Remote sensing measurements and soundings		

James Szykman, US EPA	Vaisala CL51	Mixed layer height
Thomas McGee, NASA GSFC	GSFC TROPospheric OZone DIfferential Absorption Lidar (TROPOZ DIAL)	Lidar ozone profile
Anne Thompson, NASA GSFC	iMet-1-RSB Radiosondes, DMT/EN-SCI electrochemical concentration cell (ECC) ozonesondes	O_3_, T, P, RH, WS, WD soundings

**Table 3.** Other ground and ship-based measurements. DOI: https://doi.org/10.1525/elementa.2020.00163.t3

Investigator, Institution	Instrument Name/Technique	Species/Parameters Measured
Bulkwang Supersite (Lat/Lon: 37.6098/126.9348)		

Hye-Jung Shin	Scanning mobility particle sizer (SMPS), aerodynamic particle sizer (APS), nephelometer, BAM 1020, X-ray fluorescence (XRF), ambient ion monitor (AIM), Sunset semi-continuous organic carbon/elemental carbon analyzer (SOCEC), Aethalometer	Particle size distribution, scattering, particulate matter (PM_2.5_), PM_10_, trace metals, soluble ions, organic carbon (OC), elemental carbon (EC), black carbon (BC)

Bangnyung Supersite (Lat/Lon: 37.963/124.644)		

Jinseok Han, Anyang University	High-efficiency planar diffusion scrubber-ion chromatography (HEDS-IC)	Trimethylamine (TMA), NH3
Meehye Lee, Korea University	Luminol-gas chromatography (GC)	PAN
Mindo Lee, NIER	Teledyne gas analyzers, Varian NL/450GC SMPS, APS, nephelometer, BAM 1020, XRF, AIM, Sunset SOCEC, Aethalometer	O3, CO, NOx, SO_2_, volatile organic compounds (VOCs) Particle size distribution, scattering, PM_2.5_, PM_10_, trace metals, soluble ions, OC, EC, BC
Kitai Kang, ART PLUS Co., Ltd.	SMPS	Particle size distribution

Daejeon Supersite (Lat/Lon: 36.35/127.38)		

Jeong Ah Yu, NIER	Nephelometer, BAM 1020, XRF, AIM, Sunset SOCEC, Aethalometer	Scattering, PM_2.5_, PM_10_, trace metals, soluble ions, OC, EC, BC

Gwangju Supersite (Lat/Lon: 35.2278/126.8428)		

Cheol-Soo Lim, NIER	Nephelometer, BAM 1020, XRF, AIM, Sunset SOCEC, Aethalometer	Scattering, PM_2.5_, PM_10_, trace metals, soluble ions, OC, EC, BC
Kihong Park,GwangjuInstitute of Science and Technology (GIST)	SMPS, OPC, quadrupole aerosol mass spectrometer (QAMS)	Particle size distribution, submicron chemical composition

Ulsan Supersite (Lat/Lon: 35.53/129.31)		

Mikyung Park, NIER	Teledyne gas analyzers, nephelometer, BAM 1020, XRF, AIM, Sunset SOCEC, Aethalometer	NOy, NH_3_, scattering, PM_2.5_, PM_10_, trace metals, soluble ions, OC, EC, absorption

Jeju Supersite (Lat/Lon: 33.32/126.40)		

Soojin Ban, NIER	SMPS, APS, nephelometer, BAM 1020, XRF, AIM, Sunset SOCEC, Aethalometer	Particle size distribution, scattering, PM_2.5_, PM_10_, trace metals, soluble ions, OC, EC, absorption

Seoul National University (SNU; Lat/Lon: 37.458/126.951)		

Sang-Woo Kim, SNU	Mie Scattering Lidar	Aerosol backscatter and depolarization
Robert Holz, U. Wisconsin	High spectral resolution lidar	

Korea Institute of Science and Technology (KIST) (Lat/Lon: 37.6015/127.0452)		

Hwajin Kim, KIST	High-resolution time-of-flight aerosol mass spectrometer	Chemically speciated submicron nonrefractory particulate mass and size distribution

Hankuk University of Foreign Studies (HUFS) (Lat/Lon: 37.339/127.266)		

Young Sung Ghim, HUFS	SMPS, OPC, multiangle absorption photometer (MAAP)	Particle size distribution, PM_10_, PM_2.5_, PM_1_, BC

Yonsei University (Lat/Lon: 37.564/126.935)		

Jinkyu Hong, Yonsei U.	Picarro CRDS, CL-31	CO_2_, CH_4_, boundary layer (BL) height

Jungnang (Lat/Lon: 37.5906/127.0794)		

Moon-Soo Park, HUFS	CL51, Automatic Weather Station	Aerosol backscatter, BL height, Meteorology: T, P, RH, precip, WS, WD, radiation

Gosan (Lat/Lon: 33.292/126.162)		

Sang-Woo Kim, SNU	SMPS, nephelometer, aethalometer, Mie scattering lidar	Particle size distribution, scattering, BC, aerosol backscatter and depolarization

Pyeongtaek power plant (Lat/Lon: 36.865/126.215)		

Young J. Kim, GIST	Mini MAX-DOAS	SO_2_

Fukue Island (Lat/Lon: 32.75/126.68)		

Yugo Kanaya, Japan Agency for Marine-Earth Science and Technology (JAMSTEC)	Thermo analyzers (49C, 48C, MAAP 5012, SHARP 5030), COSMOS, Metcon spectroradiometer	O_3_, CO, Black carbon, j(NO_2_), j(O^1^D)

RV Onnuri		

Carolyn Jordan, National Institute of Aerospace (NIA)	Brechtel TAP, Airphoton IN101 nephelometer, SpEx, filter sampling, MicroTOPS II	In situ aerosol absorption and scattering, spectral aerosol extinction (300–700 nm), aerosol composition and spectral optical properties, totalcolumn multiwavelength aerosol optical depth
Anne Thompson, NASA Goddard Space Flight Center (NASA GSFC)	Thermo analyzers (49C, 48C, 42C-Y), Aerodyne CAPS	O_3_, CO, NO, NOy, NO_2_
Wonkook Kim, Korea Institute of Ocean Science and Technology (KIOST)	RV Onnuri onboard automatic weather station (AWS)	Meteorology: T, P, RH, WS, WD, solar radiation, precipitation

RV Jang Mok		

James Flynn, University of Houston	Thermo analyzers (49i, 42i-TL, 48i-TLE, 43i-TL)	O_3_, NO, NO_2_, CO, SO_2_
Young-Je Park, KIOST	RV Jang Mok onboard AWS	Met: T, P, RH, WD, WS

RV Kisang		

Meehye Lee, Korea University	Thermo analyzers (49C, 48C), KENTEK Mezus-110, Luminol-GC, nephelometer, aethalometer	O_3_, CO, SO_2_, NO_2_, PAN, aerosol scattering, BC

**Table 4.** Locations of AERONET sunphotometers and Pandora spectrometers. DOI: https://doi.org/10.1525/elementa.2020.00163.t4

Site Name	Latitude	Longitude	AERONET	Pandora
Anmyeon	36.539	126.330	L	K
Baengnyeong	37.963	124.644	L	
Busan	35.235	129.083	L	K
Daegwallyeong	37.687	128.759	K	
Gangneung	37.771	128.867	L	
Gosan	33.292	126.162	L	
Gwangju	35.228	126.843	L	K
Hankuk	37.339	127.266	L	
Iksan	35.962	127.005	K	
Kyungpook	35.890	128.606	K	
Mokpo	34.913	126.437	K	
National Institute of Environmental Research	37.569	126.640	K	
Olympic Park	37.522	127.124	K	K
Seoul	37.458	126.951	L	
Songchon (Baeksa)	37.412	127.569	K	K
Taehwa	37.312	127.310	K	K
Ulsan National Institute of Science and Technology	35.582	129.190	K	
Yeoju	37.338	127.489	K	K
Yonsei	37.564	126.935	L	K

K = KORUS-AQ site; L = Long-term site.

**Table 5.** Airborne instrumentation onboard the NASA DC-8, NASA King Air, and Hanseo University King Air research aircraft. DOI: https://doi.org/10.1525/elementa.2020.00163.t5

Investigator, Institution	Instrument Name/Technique	Species/Parameters Measured
NASA DC-8 trace gas measurements		

Andrew Weinheimer, National Center for Atmospheric Research (NCAR)	NCAR 4-Channel chemiluminescence instrument	O_3_, NO, NO_2_, NOy
Glenn Diskin, NASA Langley	Diode laser spectrometer (Differential Absorption Carbon monOxide Measurement, DACOM)	CO, CH_4_, N_2_O
Glenn Diskin, NASA Langley	Diode Laser Hygrometer (DLH)	H_2_O(v)
Joshua DiGangi, NASA Langley	Nondispersive infrared spectrometer	CO_2_
Donald Blake, University of California, Irvine	Whole Air Sampler (WAS)	C_2_–C_10_ alkanes, C_2_–C_4_ alkenes, C_6_–C_9_ aromatics, C_1_–C_5_ alkylnitrates, C_1_–C_2_ halocarbons, isoprene, monoterpenes, 1,3-butadiene, carbonyl sulfide (OCS), dimethylsulfide (DMS)
Alan Fried, University of Colorado, Boulder	Compact Atmospheric Multi-species Spectrometer (CAMS)	CH_2_O, C_2_H_6_
L. Gregory Huey, Georgia Institute of Technology	Georgia Tech–Chemical Ionization Mass Spectrometer (GT-CIMS)	Peroxyacetylnitrate (PAN), peroxypropionylnitrate (PPN), peroxyacryloylnitrate (APAN), peroxybenzoylnitrate (PBZN), SO_2_, HCl
William Brune, Penn State	Airborne Tropospheric Hydrogen Oxides Sensor (ATHOS)	OH, HO_2_, OH reactivity
Ronald Cohen, University of California, Berkeley	Thermal Dissociation–Laser-Induced Fluorescence (TD-LIF)	NO_2_, sum of peroxy nitrates, sum of alkyl nitrates, aerosol-phase organic nitrates
Saewung Kim, University of California, Irvine	Chemical Ionization Mass Spectrometer (CIMS)	ClNO_2_, Cl_2_
Kyung-Eun Min, Gwangju Institute of Science and Technology	CAvity-Enhanced absorption Spectrometer for Atmospheric Research (CAESAR)	NO_2_, CHOCHO
Jeong-Hoo Park, NIER	Proton TRansfer, High Resolution, Time-of-Flight, Mass Spectrometer (PTR-HR-ToF-MS)	Toluene
Paul Wennberg, California Institute of Technology	Caltech CIMS (CIT-CIMS)	HNO_3_, HCN, H_2_O_2_, organic peroxides, organic nitrates, organic hydroxynitrates, peroxyacetic acid, cresol, glycoaldehyde
Armin Wisthaler, University of Oslo	Proton TRansfer Time-of-Flight Mass Spectrometer (PTR-ToF-MS)	Methanol, acetonitrile, acetone, methyl ethyl ketone, acetaldehyde, benzene, toluene, C8alkylbenzenes, isoprene, isoprene oxidation products, monoterpenes

NASA DC-8 aerosol measurements		

Bruce Anderson, NASA Langley	Langley Aerosol Research Group Experiment (LARGE)	Aerosol number, size distribution, optical and microphysical properties
Jack Dibb, University of New Hampshire	Soluble Acidic Gases and Aerosol (SAGA)	Bulk aerosol ionic composition, fine aerosol sulfate, HNO_3_ (and submicron NO_3_ aerosol)
Jose Jimenez, University of Colorado-Boulder	High-Resolution Time-of-Flight Aerosol Mass Spectrometer (HRToF-AMS)	Chemically-speciated submicron non-refractory particulate mass and size distribution
Taehyoung Lee, Hankuk University of Foreign Studies	Aerosol Mass Spectrometer (AMS)	Chemically speciated submicron non-refractory particulate mass
Joshua Schwarz, National Oceanic and Atmospheric Administration (NOAA)	Humidified Dual Single Particle Soot Photometer (HD-SP2)	Black carbon (BC) concentration, size distribution, mixing state, and hygroscopicity of BC containing particles
Seong Soo Yum, Yonsei University	CPC3010, DMT Cloud Condensation Nuclei Counter (CCNC)	Aerosol number, cloud condensation nuclei concentration (0.6% supersaturation)

NASA DC-8 remote sensing measurements		

Samuel Hall, NCAR	Charge-coupled device (CCD) Actinic Flux Spectrometers (CAFS)	4-*π* sr Actinic flux and derived photolysis frequencies
John Hair, NASA Langley	Differential Absorption Lidar and High Spectral Resolution Lidar (DIAL/HSRL)	Zenith and Nadir O_3_; aerosol backscatter, depolarization, extinction, and other retrieved aerosol parameters
Jens Redemann, NASA Ames	Spectrometers for Sky-Scanning, SunTracking Atmospheric Research (4STAR)	Zenith measurements of aerosol optical depth; column water vapor, O_3_, and NO_2_

NASA King Air remote sensing measurements		

Scott Janz, NASA Goddard	Geostationary Trace gas and Aerosol Sensor Optimization (GeoTASO)	Nadir column densities of NO_2_ and CH_2_O

Hanseo University King Air trace gas measurements		

Jinsoo Park, NIER	Teledyne API T400 AeroLazer AL5002 LGR GGS 24 EP Thermo TEI 43i	O_3_ CO CH_4_, CO_2_, H_2_O SO_2_
Tom Hanisco, NASA Goddard	Compact Airborne Formaldehyde Experiment (CAFÉ)	CH_2_O

**Table 6.** Air quality modeling and forecasting groups. DOI: https://doi.org/10.1525/elementa.2020.00163.t6

Investigator, Institution	Model Name/Role	Domain/Resolution
Rokjin Park, Seoul National University	GRIMs-Chem	70–15°E, 15–55°N/0.25° × 0.3125° (approximately 27 km over Korea)
Louisa Emmons, National Center for Atmospheric Research (NCAR)	CAM-Chem	Global/0.9°× 1.25°
	WRF-Tracer	East Asia/15 km, Korea/3 km
Chul Han Song, Gwangju Institute of Science and Technology	WRF-CMAQ	100–145°E, 20–45°N/15 km
Arlindo DaSilva, NASA Goddard Space Flight Center (NASA GSFC)	GEOS-5	Global/12.5 km
Gregory Carmichael, University of Iowa	WRF-Chem	East Asia/20 km, Korea/4 km
Cheol-Hee Kim, Pusan National University	WRF-Chem	East Asia/27 km
Soontae Kim, Ajou University	WRF-CAMx	Flexi-nesting 27 km/9 km/3 km
Jung-Hun Woo, Konkuk University	KU-CREATE emissions database	
David Peterson, Naval Research Laboratory (NRL) and Sang-Ok Han, National Institute of Meteorological Sciences (NIMS)	Regional Weather Forecasting	
